# Analysis of tetrahydroisoquinolines formed after simultaneous consumption of ethanol and amphetamine or its derivatives by LC–MS/MS in human blood, brain, and liver tissue

**DOI:** 10.1007/s00216-024-05540-1

**Published:** 2024-10-02

**Authors:** Marianne Sonnenberg, Constantin Czekelius, Oliver Temme, Evelyn Pawlik, Thomas Daldrup

**Affiliations:** 1 Institute of Legal Medicine, University Hospital Düsseldorf, Moorenstraße 5, 40225 Duesseldorf, Germany; 2 Institute of Organic Chemistry and Macromolecular Chemistry, Heinrich-Heine-University Duesseldorf, Universitätsstraße 1, 40225 Duesseldorf, Germany

**Keywords:** Tetrahydroisoquinoline, LC–MS/MS, Amphetamine, Brain, Liver, Blood

## Abstract

The effects of the simultaneous consumption of amphetamine or amphetamine derivatives and alcohol have not yet been adequately clarified, particularly concerning potential condensation products resulting from the endogenous reaction between these substances and their metabolites (e.g., acetaldehyde, a metabolite of ethanol). In this study, we developed an LC–MS/MS method employing liquid–liquid extraction for the qualitative detection of some relevant condensation products belonging to the class of tetrahydroisoquinolines and their derivatives in human blood, brain, and liver samples. This includes the analysis of the substrates amphetamine, methamphetamine, methylenedioxymethamphetamine, methylenedioxyamphetamine, as well as the condensation products 1,3-dimethyl-1,2,3,4-tetrahydroisoquinoline, *N*-methyl-1,3-dimethyl-1,2,3,4-tetrahydroisoquinoline, 1,3-dimethyl-7,8-methylenedioxy-1,2,3,4-tetrahydroisoquinoline, and *N*-methyl-1,3-dimethyl-7,8-methylenedioxy-1,2,3,4-tetrahydroisoquinoline. Therefore, the reference standards of the mentioned tetrahydroisoquinolines were synthesized in advance and the method was validated with regard to the question of the qualitative detection of these compounds. The validation parameters included selectivity, specificity, limit of detection, lower limit of quantification, recovery, matrix effects, and stability for blood, brain, and liver samples. Following the analysis of human blood and post-mortem tissue samples, evidence of the condensation product 1,3-dimethyl-1,2,3,4-tetrahydroisoquinoline originating from the interaction between amphetamine and acetaldehyde was identified in two liver samples. On the contrary, no evidence of this or other tetrahydroisoquinolines was found in the remaining tissue and serum samples.

**Graphical abstract:**

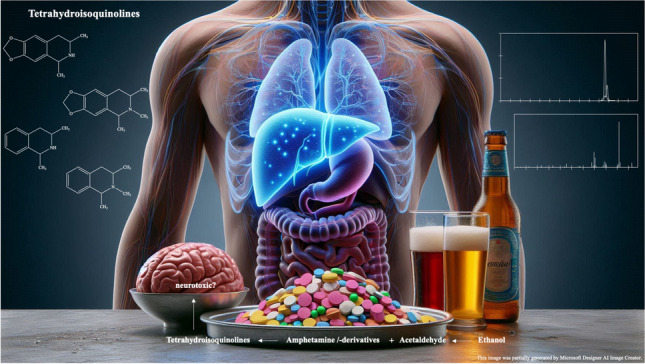

**Supplementary Information:**

The online version contains supplementary material available at 10.1007/s00216-024-05540-1.

## Introduction

The combined effect of amphetamine or amphetamine derivatives and ethanol is a subject of controversial debate [1]. While amphetamines exert central stimulant effects, ethanol primarily functions as a depressant, inhibiting the release of various neurotransmitters within the central nervous system [2]. It is difficult to assess whether the simultaneous consumption of amphetamine and alcohol results in an antagonism, an additive, or even a synergistic effect [1]. Early investigations by Todzy et al. in 1978 suggested a central antagonistic relationship, whereas subsequent studies, such as those by Makino et al. in 1990, reported a notable exacerbation of psychotic symptoms, such as heightened hallucinations and delusions [3, 4]. Furthermore, Yamamura et al. (1991) postulated that the simultaneous ingestion of alcohol may expedite the onset of methamphetamine-induced psychosis [1].

**Fig. 1:**
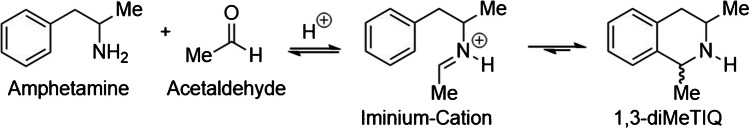
Pictet-Spengler cyclization of amphetamine and acetaldehyde to 1,3-diMe-TIQ (according to [9])

Subsequent research [1, 5, 6] have suggested a complex synergistic interaction between amphetamines and alcohol, wherein each substance modulates the effects of the other. Moreover, it seems to be a stimulant-dosis-dependent interaction [7]. Additionally, Makino et al. have postulated a neurotoxic condensation product of amphetamine and alcohol, 1,3-dimethyl-1,2,3,4-tetrahydrosioquinoline (1,3-diMeTIQ). This compound was identified in the brains and bloodstreams of chronic alcoholic rats following repeated amphetamine administration [4]. The authors documented some neurotoxic effects (tremor, curving of the back, stereotype drooling, hypersensitivity, hypertrophy of the genitals) after this treatment, akin to those observed after the single administration of 1,3-diMeTIQ. It has been suggested that this condensation product is probably enzymatically formed from amphetamine and acetaldehyde—the metabolite of ethanol—via a Pictet-Spengler ring cyclization [4, 8]. In this process, the amphetamine amino group forms an iminium ion with acetaldehyde, which subsequently undergoes electrophilic substitution at the aromatic ring to yield 1,3-diMeTIQ [9] (see Fig. 1).

**Fig. 2:**
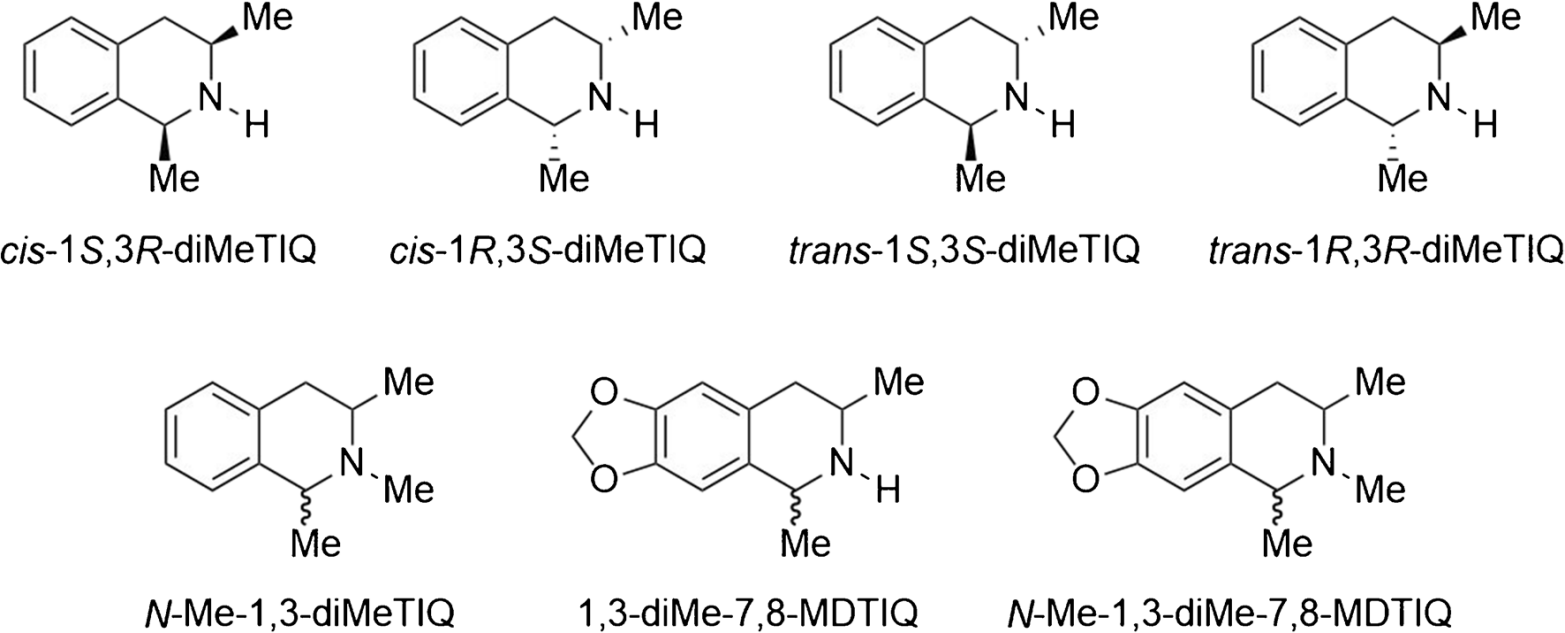
Structure of 1,3-diMeTIQ (with respective stereoisomers); *N*-Me-1,3-diMeTIQ; 1,3-diMe-7,8-MD-TIQ; and *N*-Me-1,3-diMe-7,8-MD-TIQ

The identification of this, along with other TIQs, generated through the cyclization of amphetamine derivatives like methamphetamine, methylenedioxymethamphetamine (MDMA), or methylenedioxyamphetamine (MDA), has not been reported in human samples thus far. Hence, the aim of the this study was to develop and validate a liquid chromatography-tandem mass spectrometric (LC–MS/MS) method, incorporating liquid–liquid extraction, for the analysis of amphetamine, its aforementioned derivates, and their condensation products: 1,3-diMeTIQ, *N*-methyl-1,3-dimethyl-1,2,3,4-tetrahydoisoquinoline (*N*-Me-1,3-diMeTIQ), 1,3-dimethyl-7,8-methylenedioxy-1,2,3,4-tetrahydroisoquinoline (1,3-diMe-7,8-MDTIQ), and *N*-methyl-1,3-dimethyl-7,8-methylenedioxy-1,2,3,4-tetrahydroisoquinoline (*N*-Me-1,3-diMe-7,8-MDTIQ) (refer to Fig. 2). This analytical approach encompassed the examination of human blood, brain, and liver specimens. Validation parameters included selectivity, specificity, limit of detection (LoD), lower limit of quantification (LLoQ), recovery, matrix effects, and stability in blood, brain, and liver samples, as well as linearity of the method for serum samples.

Subsequently, the presence of TIQs and their precursor substances was assessed in human blood, post-mortem brain, and liver samples.

## Material and methods

*rac*-Amphetamine sulfate was inventory of the Institute of Legal Medicine (Duesseldorf, Germany). Methamphetamine-HCl (1 mg/ml), Rac-MDA-HCl (1 mg/ml), and Rac-MDMA-HCl (1 mg/ml) were purchased from Lipomed (Herne, Germany). D_11_-Amphetamine (1 mg/ml) and D_5_-MDA (1 mg/ml) were supplied by Cerilliant (Round Rock, USA). Acetonitrile (HPLC grade and LC–MS grade), formic acid (≥ 99%, LC–MS grade), methanol (HPLC grade and LC–MS grade), and water (HPLC grade and LC–MS grade) were supplied by VWR (Radnor, USA). Methylene chloride (gradient grade), diethyl ether (p. a.), isopropanol (gradient grade), sodium hydroxide (p. a.), perchloric acid (60%, p. a.), and hydrochloric acid (25%, p. a.) were purchased from Merck (Darmstadt, Germany) and dimethyl sulfoxide (99.8%) from Fluka®—Honeywell (Offenbach, Deutschland). Ultra Turrax steel balls were obtained by IKA, Staufen, Germany.

An internal standard (ISTD) mix solution containing 1-(Me-d_3_)-TIQ-d_8_ (c = 10 ng/µl), D_11_-amphetamine (c = 5 ng/µl), and D_5_-MDA (c = 5 ng/µl) was prepared. For spiking specimen, working solutions containing 1,3-diMeTIQ, *N*-Me-1,3-diMeTIQ, 1,3-diMe-7,8-MDTIQ, *N*-Me-1,3-diMe7,8-MD TIQ, amphetamine, methamphetamine, MDA, and MDMA were prepared with 1% aqueous dimethyl sulfoxide at concentrations of 0.01 ng/µl, 0.1 ng/µl, and 1 ng/µl.

### Synthesis

All solvents and reagents used were purchased from commercial suppliers as reagent grade. They were used without further purification unless otherwise noted. Starting materials and catalysts, which were not commercially available, were synthesized by previously reported methods. Dichloromethane was dried using the Solvent Purification System MP SPS-800 by M.Braun. For reactions requiring an inert atmosphere, the glassware was dried in a compartment dryer at 120 °C, and then standard Schlenk techniques were used to work under a dry nitrogen atmosphere. Rotary evaporators combined with vacuum pumps were used for the removal of volatiles under reduced pressure. Analytical thin-layer chromatography (TLC) was performed on precoated alumina-backed silica gel plates (Macherey–Nagel, 0.2-mm thickness silica gel 60 with fluorescence indicator UV_254_), which were developed using UV fluorescence and KMnO_4_ stain solution. Flash chromatography was performed on silica gel (Macherey–Nagel, silica 60 M, 0.04–0.063 mm). ^1^H-NMR spectra were recorded on a Bruker Advance III 600 MHz or Bruker Advance III 300 MHz spectrometer. ^13^C-NMR spectra were recorded at 151 MHz. The coupling constants *J* are given in Hertz (Hz) and chemical shifts (δ) in ppm. Chemical shifts were reported in δ ppm referenced to trace amounts of chloroform δ(CHCl_3_) = 7.26 ppm in ^1^H-spectra and to the signal of deuterated chloroform δ(CDCl_3_) = 77.16 ppm in ^13^C-spectra. Multiplicities are abbreviated as s = singlet, d = doublet, t = triplet, q = quartet, m = multiplet, and br = broad signal. High-resolution mass spectra (HRMS) were performed on a Bruker Daltonics UHR-QTOF maXis 4G and compared with the calculated mass.

### Synthesis procedures

#### 1-(Methyl-d_3_)-1,2,3,4-tetrahydroisoquinoline-1,2,3,4,5,6,7,8-d_8_

1-Methylisoquinoline (1.00 g, 6.98 mM) and benzoic acid (128 mg, 1.05 mM, 15 mol%) were added to deuterium oxide (15 ml) and the mixture heated to 80 °C under nitrogen for 6 h. After addition of solid sodium hydrogen carbonate (200 mg), the mixture was extracted with diethyl ether (3 × 50 ml), the combined organic layers dried over sodium sulfate, and the solvent removed in vacuo giving the crude 1-(methyl-d_3_)-isoquinoline (1.10 g, 94% deuteration by ^1^H NMR). A part of this intermediate (250 mg, 1.71 mM) was dissolved in methanol-d_4_ (1 ml) and platinum(IV) oxide (96.6 mg, 422 μM) and acetic acid-d_4_ (107 mg, 96 μl, 3.46 mM) added under nitrogen [10]. A balloon of deuterium gas was connected to the system and the suspension stirred at rt for 3 days. The mixture was filtered and the solid rinsed with diethyl ether (20 ml). Flash column chromatography (silica gel, ethyl acetate) gave the reduced material as a colorless oil (50.0 mg, 316 μM, 18%). It was transformed into the hydrochloride salt by dissolving the free base in methanol-d_4_ (5 mL) and addition of hydrochloric acid in methanol (1.0 M, 500 μL) followed by removal of all volatiles in vacuo giving a colorless solid.

^**1**^**H NMR** (free base, CDCl_3_, 600 MHz): δ = 2.59 (m, 1H, C*H*D-N), 1.71 (m, 1H, Ar-C*H*D). Residual ^1^H signals due to incomplete deuteration: 8.15 (s), 6.84 (s), 4.68 (br. s), 2.42 (m), 2.40 (m) (s. Electronic Supplementary Information, Fig.S1).

^**13**^**C NMR** (free base, CDCl_3_, 151 MHz): δ = 156.7 (s), 146.6 (m), 144.4 (m), 131.3 (m), 122.2 (s), 122.0 (m), 29.0 (m), 25.6 (m), 22.4 (m), 21.4 (m) (s. Electronic Supplementary Information, Fig. S2).

**HRMS** (ESI, in MeOH): calculated for C_10_H_4_D_10_N: 158.1748; found 158.1746 (s. Electronic Supplementary Information, Fig. S3).

#### General procedure for the N-methylation of tetrahydroisoquinolines[11, 12]

The tetrahydroisoquinoline is dissolved in dichloromethane (c = 150 mM) and solid sodium hydrogencarbonate (4.3 equiv) added. After stirring for 5 min at rt, methylchloroformate (2.1 equiv) is added dropwise. Stirring is continued overnight at rt. Additional quantities of solid sodium hydrogencarbonate (4.3 equiv) and methylchloroformate (2.1 equiv) are added sequentially and stirring at rt is continued for 48 h. After addition of sat. aqueous NaHCO_3_ solution, stirring is continued for 3 h, and then the mixture is extracted with dichloromethane. The combined organic layers are dried over sodium sulfate and the solvent removed in vacuo. The crude carbamate is dissolved in dry tetrahydrofuran (c = 50 mM) under nitrogen and lithium aluminum hydride (3.2 equiv) added at rt. The suspension is heated to reflux for 4 h. After cooling to rt, isopropanol (1.8 equiv) and sodium hydroxide solution (10 M in water, 6.9 equiv) are added sequentially. After stirring for 1 h at rt, water is added and the mixture extracted with ethyl acetate. The combined organic layers are dried over sodium sulfate and the solvent removed in vacuo. The residue is purified by column chromatography (silica gel, eluent: dichloromethane/8% methanol/1% aq. ammonia).

#### *cis/trans*-1,2,3-Trimethyl-1,2,3,4-tetrahydroisoquinoline [13] (N-methyl-1,3-dimethyl-1,2,3,4-tetrahydoisoquinoline)

Following the procedure by Robinson, 1,2,3-trimethyl-1,2,3,4-tetrahydroisoquinoline [13] was obtained from 1,3-dimethyl-1,2,3,4-tetrahydroisoquinoline [14–16] in 87% yield as a mixture of diastereomers (s. Electronic Supplementary Information, Fig. S4 and S5).

^**1**^**H NMR** (free base, CDCl_3_, 600 MHz): δ = 7.15 – 6.96 (m, 4H), 3.92 – 3.81 (m, 1H), 3.32 – 2.78 (m, 1H), 2.78 – 2.53 (m, 2H), 2.31 (d, J = 19.6 Hz, 3H), 1.42 (dd, J = 42.0, 6.8 Hz, 3H), 1.19 (dd, J = 26.0, 6.4 Hz, 3H)) (s. Electronic Supplementary Information, Fig. S6).

The data are in agreement with the reported ones [17].

#### *cis/trans*-5,6,7-Trimethyl-5,6,7,8-tetrahydro-[1,3]dioxolo[4,5-g]isoquinoline (N-methyl-1,3-dimethyl-7,8-methylenedioxy-1,2,3,4-tetrahydroisoquinoline)

Following the general procedure for the *N*-methylation of tetrahydroisoquinolines, 5,6,7-trimethyl-5,6,7,8-tetrahydro-[1,3]dioxolo[4,5-*g*]isoquinoline was obtained from 5,7-dimethyl-5,6,7,8-tetrahydro-[1,3]dioxolo[4,5-*g*]isoquinoline [17] in 56% yield as a mixture of diastereomers.

^**1**^**H NMR** (free base, CDCl_3_, 600 MHz): *trans*-isomer: δ = 6.62 (s, 1H, arom. H), 6.50 (s, 1H, arom. H), 5.88 (s, 2H, OC*H*_2_O), 3.59 (q, *J* = 6.6 Hz, 1H, ArC*H*N), 2.45–2.75 (m, 3H, ArCH_2_ and CMe*H*N), 2.31 (s, 3H, N*Me*), 1.45 (d, *J* = 6.6 Hz, 3H, *Me*), 1.23 (d, *J* = 5.9 Hz, 3H, *Me*). *cis*-isomer: δ = 6.53 (s, 1H, arom. H), 6.50 (s, 1H, arom. H), 5.88 (s, 2H, OC*H*_2_O), 3.73 (q, *J* = 6.8 Hz, 1H, ArC*H*N), 2.45–2.75 (m, 3H, ArCH_2_ and CMe*H*N), 2.36 (s, 3H, N*Me*), 1.35 (d, *J* = 6.7 Hz, 3H, *Me*), 1.14 (d, *J* = 6.5 Hz, 3H, *Me*) (s. Electronic Supplementary Information, Fig. S9).

^**13**^**C NMR** (free base, CDCl_3_, 151 MHz): *trans*-isomer: δ = 146.0, 145.6, 132.2, 128.0, 107.8, 106.5, 100.7, 60.4, 55.1, 37.2, 36.3, 22.1, 20.4. *cis*-isomer: δ = 145.8, 145.8, 132.4, 126.6, 108.4, 107.0, 100.6, 58.9, 47.9, 37.2, 33.8, 20.4, 17.8 (s. Electronic Supplementary Information, Fig. S10).

**HRMS** (ESI, in MeOH): calculated for C_13_H_18_NO_2_: 220.1332; found 220.1334.

### Sample specimen

Post-mortem brain and liver samples as well as serum samples of living subjects testing positive for amphetamine, amphetamine derivatives, and ethanol were obtained from the Institutes of Legal Medicine Bonn (one brain sample), Duesseldorf (six brain and liver samples, 42 serum samples), Essen (eight brain and liver samples), Cologne (nine brain and six liver samples), and Rostock (three brain and five liver samples). Sample data were anonymized prior to analysis. For method validation, brain and liver samples, collected during autopsies at the Institute of Legal Medicine Duesseldorf, as well as serum tested negative for the aforementioned TIQs, amphetamine, and amphetamine derivatives, were used for all relevant parameters. Pig brain was used as matrix for calibration in tissue. All samples were stored at − 20 °C and temporarily at room temperature.

### Analysis of human brain, liver, and serum samples

Brain and liver samples were extracted according to Kohno et al. (1986) [18] and Hara et al. (2009) [19].

#### Homogenization of liver samples

Five hundred milligrams of wet liver tissue was initially cut into coarse pieces and mixed with 1 ml hydrochloric acid (0.1 M) and the ISTD mix. The sample was incubated at 70 °C for 1 h in a sealed 10-ml vial. Subsequently, the softened tissue was transferred into a 2-ml reaction tube and cut once more crudely, and an IKA steel ball was added. The sample was mixed for 10 min and centrifuged at 14,000 rpm for 10 min. The crude supernatant fraction of the homogenate was separated from the pellet. The pellet was mixed with 400 µl hydrochloric acid (0.1 M) for 10 min once again. After repeated centrifugation, the crude supernatant fraction of the homogenate and the hydrochloric acid supernatant was combined.

#### Homogenization of brain samples

Five hundred milligrams of brain tissue was homogenized after the addition of 10 µl ISTD mix, an IKA steel ball, and 500 µl perchloric acid (0.4 M). The brain sample was mixed for 10 min and centrifuged at 14,000 rpm for 10 min. The crude supernatant fraction of the homogenate was separated from the pellet. This pellet was mixed with 400 µl perchloric acid (0.4 M) for 10 min once again. After repeated centrifugation, the crude supernatant fraction of the homogenate supernatant and the perchloric acid supernatant was combined.

#### Extraction of brain and liver samples

Each sample was washed with 900 µl diethyl ether and extracted with 100 µl isopropanol, 500 µl methylene chloride, and 75 µl aqueous sodium hydroxide (6 M) for 10 min. After centrifugation at 14,000 rpm for 10 min, the aqueous phase was separated and extracted again with methylene chloride. The extracts were combined and mixed with 900 µl hydrochloric acid (0.1 M) for 10 min. After centrifugation, the aqueous acidic phase was treated with 75 µl aqueous sodium hydroxide and 500 µl methylene chloride for 10 min. The organic extracts were separated once again after centrifugation (14,000 rpm for 10 min). Before the sample was evaporated to dryness under a stream of nitrogen, 20 µl of methanolic hydrochloric acid (0.1 M) was added. The residue was resuspended in 60 µl water/methanol (95/5 v/v) containing 0.1% formic acid.

#### Extraction of serum samples

Five hundred microliters of serum was extracted with 500 µl of a mixture of methylene chloride and diethyl ether (70/30, v/v) after addition of 10 µl ISTD mixture and 50 µl aqueous sodium hydroxide solution (2 N). The extract was evaporated to dryness and reconstituted according to the brain and liver samples.

### Instrumental analysis

A Waters (Milford, USA) Acquity UPLC system with a TQ detector and an ACE® Excel™ C18-AR HPLC column (2.1 × 150 mm, 2 µm particle size) in combination with an EXL-PCF05 ACE®, UHPLC pre-column filter (0.5 µm, Titanium Frit) was used. Ten microliters of the sample extract was injected in a partial loop overfill injection mode. Column temperature was set to 60 °C and the flow rate to 0.55 ml/min. The mobile phase A consisted of water with 0.1% formic acid and the mobile phase B of methanol with 0.1% formic acid. Table 1 shows the elution gradient.

**Table 1:** The elution gradient used for the LC–MS/MS analysis of TIQs, amphetamine, and amphetamine derivatives

Total time in minutes	Mobile phase A in %	Mobile phase B in %
Initial	95	5
4.00	71	29
7.50	50	50
8.25	5	95
8.75	5	95
9.00	95	5
11.50	95	5

Mobile phase A, 0.1% formic acid in water; mobile phase B, 0.1% formic acid in methanol.

Mass spectrometry (MS) was performed in a positive electrospray ionization multiple reaction monitoring mode (MRM). The following MS-parameters were adjusted: source temperature, 150 °C; desolvation temperature, 400 °C; desolvation gas flow, 800 l/h; cone gas flow, 20 l/h; collision gas flow, 0.2 ml/min. Two ion transitions were monitored for each analyte (see Table 2). For quantification of amphetamine and methamphetamine, D_11_-amphetamine was used as the ISTD, while D_5_-MDA served as the ISTD for the quantification of MDA and MDMA and 1-(Me-d_3_)-TIQ-d_8_ for the quantification of the TIQs.

**Table 2:** Retention time in minutes, ion transition, qualifier to target ratio in %, cone and collision energy in volt (V)

Analyte	Retention time in minutes	Ion transition m/z	Qualifier to target ratio in %	Cone energy in V	Collision energy in V
1-(Me-d_3_)-TIQ-d_8_	3.68	156.0 > 96.0 **156.0 > 126.0**	10	35 35	30 25
1,3-diMeTIQ	3.89/4.15^a^	162.0 > 117.0 **162.0 > 145.0**	50/49^a^	28 28	22 12
1,3-diMe-7,8-MDTIQ	4.24/4.57^a^	206.0 > 105.0 **206.0 > 163.0**	17/15^a^	28 28	24 15
*N*-Me-1,3-diMeTIQ	4.28	176.0 > 117.0 **176.0 > 145.0**	57	28 28	20 14
*N*-Me-1,3-diMe-7,8-MDTIQ	4.62	220.0 > 131.0 **220.0 > 163.0**	9	26 26	25 12
D_11_-amphetamine	3.29	147.3 > 130.2 **147.3 > 98.2**	61	15 15	8 23
Amphetamine	3.41	136.1 > 119.1 **136.1 > 91.1**	115	15 15	8 23
Methamphetamine	3.78	150.1 > 119.1 **150.1 > 91.1**	47	20 20	10 12
D_5_-MDA	3.78	185.1 > 138.1 **185.1 > 168.1**	24	22 22	18 10
MDA	3.83	180.1 > 133.1 **180.1 > 163.1**	17	22 22	18 10
MDMA	4.15	194.1 > 133.1 **194.1 > 163.0**	31	20 20	20 14

^a^Retention times and ion transitions of respective trans- and cis-diastereomers; target transition in bold

### Validation

Method validation for serum, brain, and liver tissue analysis adhered to the criteria outlined by the German Society of Toxicological and Forensic Chemistry (GTFCh) [20]. Evaluation of the samples was carried out using the software Valistad 2.0 (Arvecon GmbH, Walldorf, Germany).

#### Selectivity

To assess selectivity, six blank brain, liver, and serum samples devoid of ISTD addition, along with two blank samples per specimen with ISTD, were examined to identify any interfering signals.

#### Limit of detection and lower limit of quantification

Determination of the limit of detection and lower limit of quantification involved constructing calibration curves within defined concentration ranges for each specimen (brain, 0.5 to 25 ng/g; serum, 0.1 to 5.0 ng/ml; liver, 0.75 to 12.5 ng/g) according to the criteria of Valistat 2.0.

#### Matrix effects and recovery

Matrix effects and recoveries were evaluated using six control samples of analytical standard solution, six spiked matrix samples, and six spiked extracts at concentrations near the lower limit of quantification (c (brain) = 4 ng/g; c (liver) = 4 ng/g; c (serum) = 1 ng/g). Matrix effects were determined by the ratio of absolute areas of spiked extracts to control samples, while recovery was assessed by the ratio of spiked matrix to spiked extracts.

#### Stability

Processed sample stability was established by analyzing six spiked and pooled brain (c = 4 ng/g), liver (c = 4 ng/g), and serum (c = 1 ng/g) samples, aliquoted post-extraction over 5 h (brain and liver) and 8.5 h (serum).

Freeze–thaw stability was estimated using six spiked stability samples subjected to multiple freeze–thaw cycles (brain samples, three cycles over 3 days; liver samples, three cycles over 3 weeks; serum samples, four cycles over 2 weeks). Thereby, the samples were frozen at − 20 °C and subsequently thawed at ambient temperature for a duration of 1 h per cycle. Furthermore, six control samples were spiked and assessed directly without exposure to any freeze–thaw cycle. The selection of analyte concentrations in the brain, liver, and serum mirrored the stability conditions of the processed sample.

Long-term stability was determined by storing brain samples (*n* = 6) at − 20 °C for 100 days, liver samples for 53 days, and serum samples for 65 days prior to analysis. Additionally, six spiked control samples were analyzed without any treatment. Analyte concentrations mirrored those used for processed sample stability.

Furthermore, stability samples lacking substance addition (blank stability samples) were analyzed to check for interfering signals that could arise during the freeze–thaw or the long-term stability treatment. Hence, three control samples devoid of any treatment, alongside three samples, subjected to freeze–thaw cycles or prolonged storage, were evaluated.

#### Quantification

To assess linearity, ten replicated serum samples were spiked with amphetamine, methamphetamine, MDA, and MDMA at six different concentrations ranging from 25 to 750 ng/ml.

For quantification of amphetamine and its derivatives in tissue samples, calibration was conducted using brain samples with pig brain as blank matrix. Duplicates were spiked in seven or eight different concentrations ranging between 25 and 500 ng/g.

## Results

**Fig. 3:**
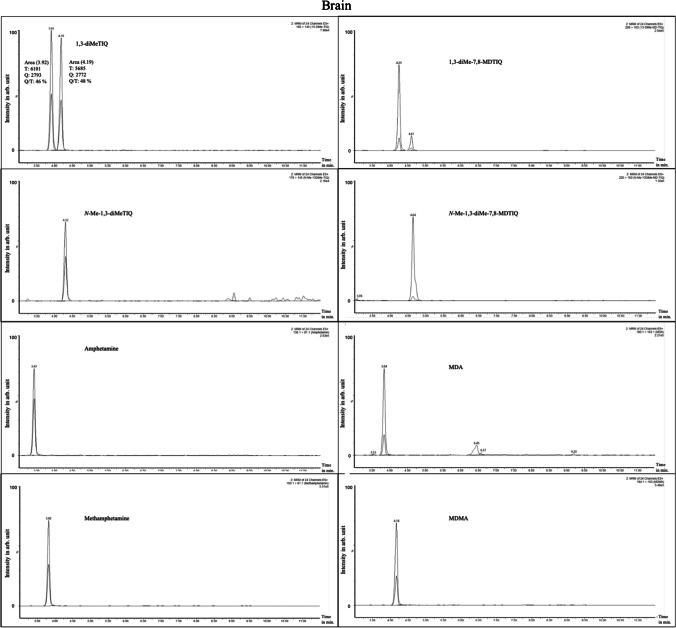
Extracted ion chromatograms (time in minutes vs. intensity in arbitrary (arb.) units in %) of human brain tissue (c = 4 ng/g) after fluid–fluid-extraction. For each compound, target- and qualifier-transitions are shown (from left to right and from top to bottom): *trans/cis-*1,3-diMeTIQ (t_R_ = 3.92 min and 4.19 min exemplary with respective areas for each mass transition); *N*-Me-1,3-diMeTIQ (t_R_ = 4.32 min); *trans/cis-*1,3-diMe-7,8-MDTIQ (t_R_ = 4.25 min and 4.61 min); *N*-Me-1,3-diMe-7,8-MDTIQ (t_R_ = 4.64 min); amphetamine (t_R_ = 3.43 min); methamphetamine (t_R_ = 3.82 min); MDA (t_R_ = 3.84 min); MDMA (t_R_ = 4.18 min)

For the analysis of 26 brain, 28 liver, and 42 serum samples, an LC–MS/MS method employing fluid–fluid-extraction was validated for the detection of 1,3-diMeTIQ, *N*-Me-1,3-diMeTIQ, 1,3-diMe-7,8-MDTIQ, and *N*-Me-1,3-diMe-7,8-MDTIQ, alongside their substrates amphetamine, methamphetamine, MDA, and MDMA. Figure 3 illustrates the LC–MS/MS extracted ion MRM chromatograms of each compound of a spiked brain sample at a concentration of 4 ng/g.

**Fig. 4:**
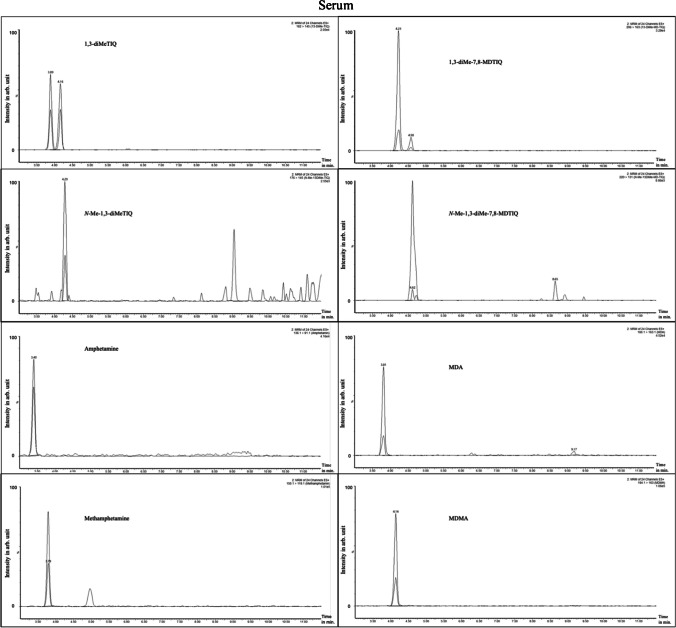
Extracted ion chromatograms (time in minutes vs. intensity in arbitrary (arb.) units in %) of human serum (c = 1 ng/ml) after fluid–fluid-extraction. For each compound, target- and qualifier-transitions are shown (from left to right and from top to bottom): *trans/cis*-1,3-diMeTIQ (t_R_ = 3.89 min and 4.16 min); *N*-Me-1,3-diMeTIQ (t_R_ = 4.29 min); *trans/cis*-1,3-diMe-7,8-MDTIQ (t_R_ = 4.23 min and 4.58 min); *N*-Me-1,3-diMe-7,8-MDTIQ (t_R_ = 4.62 min); amphetamine (t_R_ = 3.40 min); methamphetamine (t_R_ = 3.79 min); MDA (t_R_ = 3.81 min); MDMA (t_R_ = 4.14 min)

**Fig. 5:**
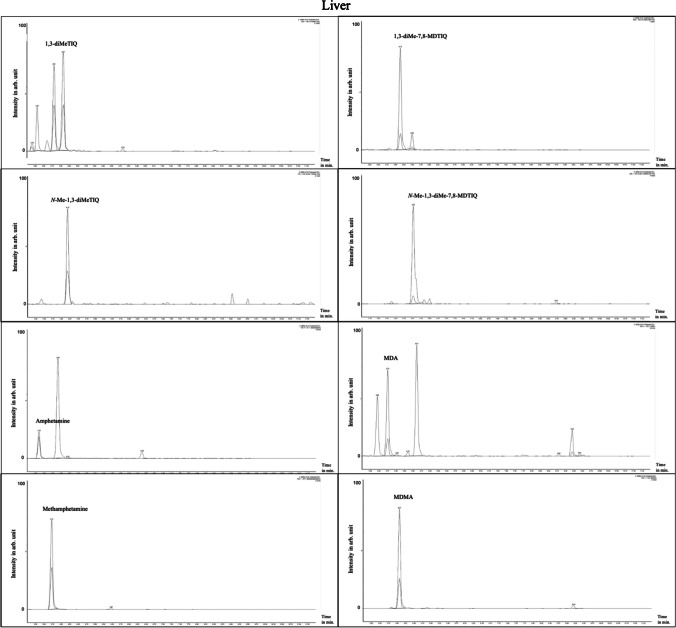
Extracted ion chromatograms (time in minutes vs. intensity in arbitrary (arb.) units in %) of human liver (c = 4 ng/ml) after fluid–fluid-extraction. For each compound, target- and qualifier-transitions are shown (from left to right and from top to bottom): *trans/cis*-1,3-diMeTIQ (t_R_ = 3.81 min and 4.07 min); *N*-Me-1,3-diMeTIQ (t_R_ = 4.19 min); *trans/cis*-1,3-diMe-7,8-MDTIQ (t_R_ = 4.15 min and 4.49 min); *N*-Me-1,3-diMe-7,8-MDTIQ (t_R_ = 4.52 min); amphetamine (t_R_ = 3.35 min); methamphetamine (t_R_ = 3.72 min); MDA (t_R_ = 3.76 min); MDMA (t_R_ = 4.07 min)

The *trans*- and *cis*-isomers of 1,3-diMeTIQ (t_R_ = 3.92 min and t_R_ = 4.19 min) and of 1,3-diMe-7,8-MDTIQ (t_R_ = 4.25 min and t_R_ = 4.61 min) were successfully separated. However, the diastereomers of *N*-Me-1,3-diMeTIQ and *N*-Me-1,3-diMe-7,8-MDTIQ could not be distinguished and were measured as a single peak. The ratio of *trans*- and *cis*-1,3-diMeTIQ is nearly equal, while *trans*-1,3-diMe-7,8-MDTIQ (peak (t_R_ = 4.25 min) = 83%) exhibited a higher proportion than the corresponding *cis*-1,3-diMe-7,8-MDTIQ (peak (t_R_ = 4.61 min) = 17%) (see Fig. 3). Furthermore, Figs. 4 and 5 show extracted ion chromatograms of each analyte in spiked human serum and in human liver tissue.

### Validation

#### Selectivity

**Fig. 6:**
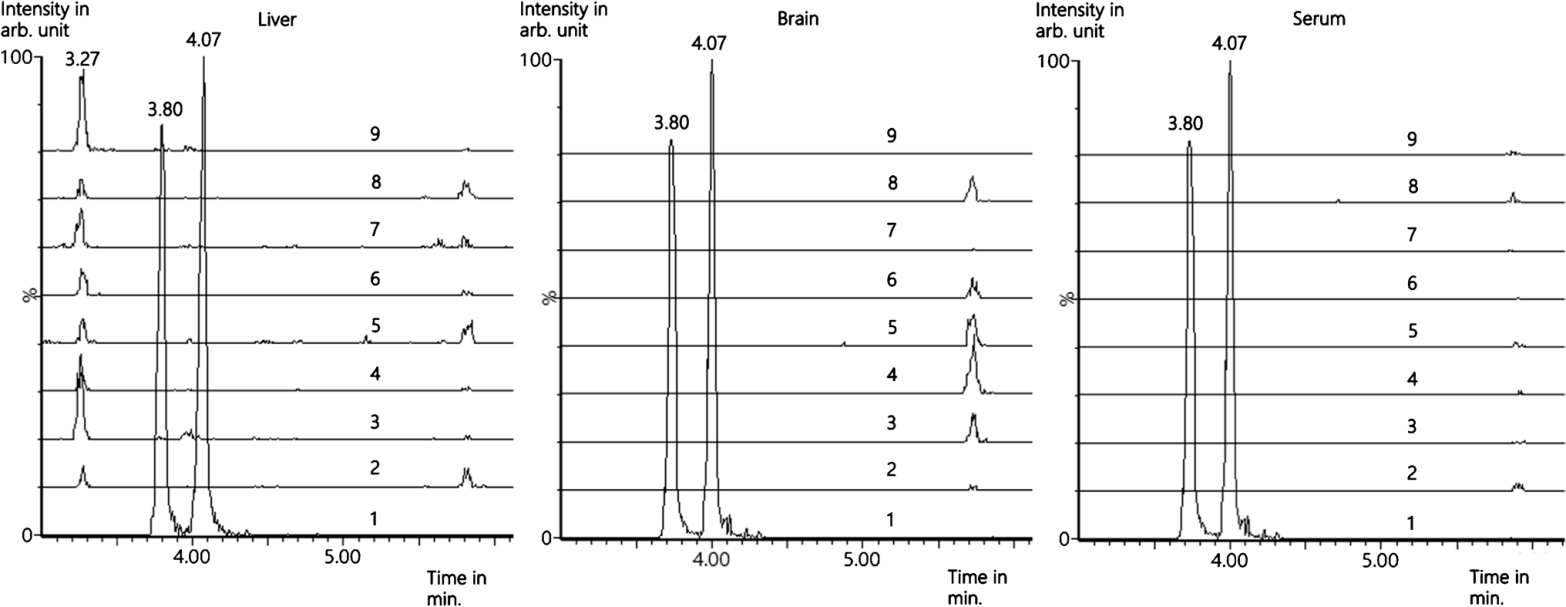
Extracted ion chromatograms (time in minutes vs. intensity in arbitrary (arb.) units in %) with an incremental offset of 10%) of selectivity measurements in human liver (left), human brain (middle), and human serum (right) after fluid–fluid-extraction for the MRM-target-transition 162 > 145 of *trans/cis*-1,3-diMeTIQ (t_R_ = 3.80 min and 4.07 min). Sample 1, reference substance; samples 2 to 7, blank samples (matrix devoid of ISTD addition); samples 8 and 9, blank samples with ISTD. The peak at 3.27 min seems to be tryptamine; as a result of putrefaction, all other signals were matrix induced

No interfering signals were observed in brain, liver, and serum samples (see Fig. 6).

#### Limit of detection and lower limit of quantification

The lowest LoDs and LLoQs were identified in serum (LoD, 0.1 ng/ml; LLoQ, 0.2 ng/ml). In brain tissue, LoD and LLoQ ranged from 0.3 to 2.2 ng/g, and in liver tissue from 0.1 to 4.5 ng/g (see Table 3).

**Table 3:** Limit of detection (LoD) and lower limit of quantification (LLoQ) of the TIQs and amphetamines in serum (in ng/ml), brain, and liver tissue (in ng/g) (determination by extrapolation of a calibration curve with five concentration levels using Valistat 2.0)

	Brain		Liver		Serum	
Analyte	LoD in ng/g	LLoQ in ng/g	LoD in ng/g	LLoQ in ng/g	LoD in ng/ml	LLoQ in ng/ml
*trans*-1,3-diMeTIQ	0.3	2.2	0.7	3.4	0.1	0.5
*cis*-1,3-diMeTIQ	0.3	1.2	0.1	0.9	0.2	0.6
*trans*-1,3-diMe-7,8-MDTIQ	0.4	1.6	1.3	1.5	0.1	0.2
*cis*-1,3-diMe-7,8-MDTIQ	2.2	5.7	4.5	4.5	0.3	1.2
*N*-Me-1,3-diMeTIQ	1.3	3.6	1.2	2.2	0.3	0.6
*N*-Me-1,3-diMe-7,8-MDTIQ	1.1	1.9	1.2	6.3	0.5	2.0
Amphetamine	0.5	1.5	0.4	1.0	0.1	0.4
Methamphetamine	0.6	1.1	0.6	1.5	0.1	0.3
MDA	0.7	0.8	0.6	3.0	0.1	0.4
MDMA	0.4	1.9	0.4	1.2	0.1	0.3

#### Matrix effects and recovery

The results for matrix effects and recoveries are summarized in Table 4. All TIQs and amphetamines met the acceptance interval for matrix effects (80 to 120%) according to the guideline of the GTFCh. However, the standard deviation for amphetamine, methamphetamine, MDMA, and N-Me-1,3-diMeTIQ slightly exceeded the 25% limit (26 to 30%) in liver samples.

**Table 4:** Recoveries and matrix effects of the TIQs and amphetamines in % with standard deviation (SD), spiked brain (c = 4 ng/g), liver (c = 4 ng/g), and serum (c = 1 ng/g) samples

	Matrix effects in % (± SD)			Recoveries in % (± SD)		
Analyte	Brain c = 4 ng/g	Liver c = 4 ng/g	Serum c = 1 ng/ml	Brain c = 4 ng/g	Liver c = 4 ng/g	Serum c = 1 ng/ml
*trans*-1,3-diMeTIQ	97 ± 2.9	94 ± 25	105 ± 12	50 ± 7.1	46 ± 12	84 ± 7.3
*cis*-1,3-diMeTIQ	96 ± 7.0	108 ± 24	99 ± 12	47 ± 7.0	47 ± 10	84 ± 7.5
*trans*-1,3-diMe-7,8-MDTIQ	96 ± 7.0	80 ± 19	106 ± 10	47 ± 7.4	47 ± 10	85 ± 8.8
*cis*-1,3-diMe-7,8-MDTIQ	81 ± 17	81 ± 16	80 ± 18	48 ± 14	48 ± 9.8	79 ± 20
*N*-Me-1,3-diMeTIQ	102 ± 10	96 ± 28	116 ± 25	42 ± 9.3	51 ± 16	82 ± 20
*N*-Me-1,3-diMe-7,8-MDTIQ	100 ± 9.3	86 ± 25	78 ± 13	41 ± 5.5	43 ± 10	77 ± 12
Amphetamine	95 ± 4.2	96 ± 30	105 ± 9.1	41 ± 7.8	45 ± 10	77 ± 4.2
Methamphetamine	99 ± 4.8	100 ± 26	103 ± 6.4	53 ± 6.2	52 ± 12	85 ± 6.9
MDA	97 ± 6.2	107 ± 24	107 ± 7.0	49 ± 7.5	48 ± 9.5	82 ± 7.5
MDMA	90 ± 7.2	89 ± 26	106 ± 8.8	48 ± 7.0	48 ± 12	86 ± 6.7

Recoveries in brain and liver tissue were close to the GTFCh criterion ≥ 50% (41 to 53%), fully meeting the criterion in serum (77 to 85%). Additionally, the standard deviation was ≤ 20% in all three matrices.

#### Stability

Processed sample stability over 8.5 h in serum and 5 h in brain and liver showed minimal instabilities (see Table 5—liver tissue 9–20%; brain tissue 7–22%, serum 6–23%), with only *cis*-1,3-diMe-7,8-MDTIQ exhibiting a notable decrease in brain tissue (31%).

**Table 5:** Processed sample stability of the TIQs and amphetamines: decrease of absolute peak area in % in spiked brain (c = 4 ng/g), liver (c = 4 ng/g), and serum (c = 1 ng/g) samples, over a period of 5 h and 8.5 h, respectively (*n* = 6)

Analyte	Brain decrease in peak area in % over 5 h c = 4 ng/g	Liver decrease in peak area in % over 5 h c = 4 ng/g	Serum decrease in peak area in % over 8.5 h c = 1 ng/ml
*trans*-1,3-diMeTIQ	22	19	20
*cis*-1,3-diMeTIQ	14	14	18
*trans*-1,3-diMe-7,8-MDTIQ	22	20	6
*cis*-1,3-diMe-7,8-MDTIQ	31	17	18
*N*-Me-1,3-diMeTIQ	13	16	23
*N*-Me-1,3-diMe-7,8-MDTIQ	16	18	18
Amphetamine	16	17	10
Methamphetamine	12	14	9
MDA	7	9	12
MDMA	19	13	11

Freeze–thaw stability analysis met GTFCh guidelines, with slight deviations observed only for *cis*-1,3-diMe-7,8-MDTIQ (115% instead of 110%).

The evaluation of freeze–thaw stability was accomplished by using the area ratios of analyte and ISTD. According to the guideline of the GTFCh, the mean values of the stability samples were within the acceptance interval of 90–110% of the control samples for nine of ten analytes (see Table 6). Only the mean value of *cis*-1,3-diMe-7,8-MDTIQ was slightly outside the limit (115% instead of 110%). Additionally, it can be noted that the 90% confidence interval (90%-CI) of stability samples fulfilled the criteria of 80–120% of the mean value of the control samples in all substances.

**Table 6:** Freeze–thaw stability of the TIQs and amphetamines: mean value (MV) and 90% confidence interval (90%-CI) of stability samples (*n* = 6) in % in spiked brain (c = 4 ng/g), liver (c = 4 ng/g), and serum (c = 1 ng/g) samples, calculated from area ratios of analyte and ISTD

Analyte	Brain c = 4 ng/g		Liver c = 4 ng/g		Serum c = 1 ng/ml	
	MV in %	90%-CI in %	MV in %	90%-CI in %	MV in %	90%-CI in %
*trans*-1,3-diMeTIQ	99	[94; 103]	108	[103; 112]	101	[95; 107]
*cis*-1,3-diMeTIQ	102	[97; 108]	107	[101; 113]	100	[98; 102]
*trans*-1,3-diMe-7,8-MDTIQ	102	[98; 105]	109	[109; 113]	101	[97; 105]
*cis*-1,3-diMe-7,8-MDTIQ	100	[93; 106]	115	[109; 120]	101	[93; 109]
*N*-Me-1,3-diMeTIQ	108	[102; 114]	105	[103; 108]	104	[99; 108]
*N*-Me-1,3-diMe-7,8-MDTIQ	105	[98; 112]	109	[105; 113]	96	[91; 100]
Amphetamine	107	[103; 110]	96	[93; 98]	101	[94; 108]
Methamphetamine	110	[107; 114]	99	[96; 101]	100	[99; 102]
MDA	102	[100; 104]	97	[95; 99]	102	[99; 105]
MDMA	97	[93; 101]	102	[100; 104]	103	[101; 104]

Stability analysis of brain, liver, and serum samples without any addition of the analytes showed no indication for new substances arising from the freeze–thaw treatment.

Long-term stability analysis indicated stability for all analytes across brain, liver, and serum samples.

Estimation of long-term stability was done in an analogous fashion to the freeze–thaw stability. For liver samples, absolute areas were taken into account instead of the area ratios of analyte and ISTD, which were used for brain and serum samples. Methamphetamine was the only substance whose mean value of the stability samples in brain was outside of the lower acceptance limit of 90% (see Table 7, methamphetamine 82%) in the respective period. All other amphetamines and TIQs have shown long-term stability in brain, liver, and serum.

**Table 7:** Long-term stability of the TIQs and amphetamines: mean value (MV) and 90% confidence interval (90%-CI) of stability samples (*n* = 6) in % in spiked brain (c = 4 ng/g), liver (c = 4 ng/g), and serum (c = 1 ng/g) samples, calculated from area ratios of analyte and ISTD (brain and serum) or absolute areas (liver)

Analyte	Brain c = 4 ng/g		Liver c = 4 ng/g		Serum c = 1 ng/g	
	MV in %	90%-CI	MV in %	90%-CI	MV in %	90%-CI
*trans*-1,3-diMeTIQ	100	[97; 104]	103	[94; 112]	109	[104; 114]
*cis*-1,3-diMeTIQ	97	[91; 104]	94	[89; 98]	109	[104; 114]
*trans*-1,3-diMe-7,8-MDTIQ	104	[100; 109]	110	[107; 113]	103	[101; 104]
*cis*-1,3-diMe-7,8-MDTIQ	99	[90; 107]	101	[97; 105]	105	[99; 110]
*N*-Me-1,3-diMeTIQ	107	[98; 116]	98	[93; 102]	108	[97; 119]
*N*-Me-1,3-diMe-7,8-MDTIQ	98	[94; 102]	109	[107; 112]	105	[100; 110]
Amphetamine	92	[88; 96]	98	[94; 101]	106	[98; 114]
Methamphetamine	82	[80; 84]	104	[101; 107]	108	[105; 110]
MDA	97	[94; 100]	97	[93; 100]	107	[103; 111]
MDMA	95	[92; 98]	99	[96; 101]	105	[102; 107]

Blank stability samples, which were treated in the same way like stability samples, did not indicate any false positive result for the TIQs and amphetamines or other substances that could interfere with the analytes.

#### Quantification

**Fig. 7:**
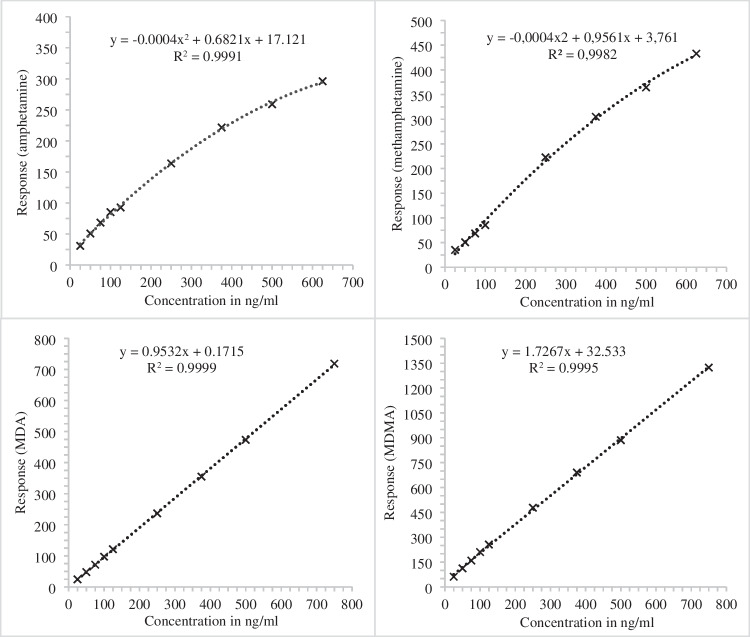
Calibration curve of amphetamine (quadratic), methamphetamine (quadratic), MDA (linear), and MDMA (linear) in serum (*n* = 2) after fluid–fluid-extraction with the respective regression coefficients (*R*^2^) and calibration functions

**Fig. 8:**
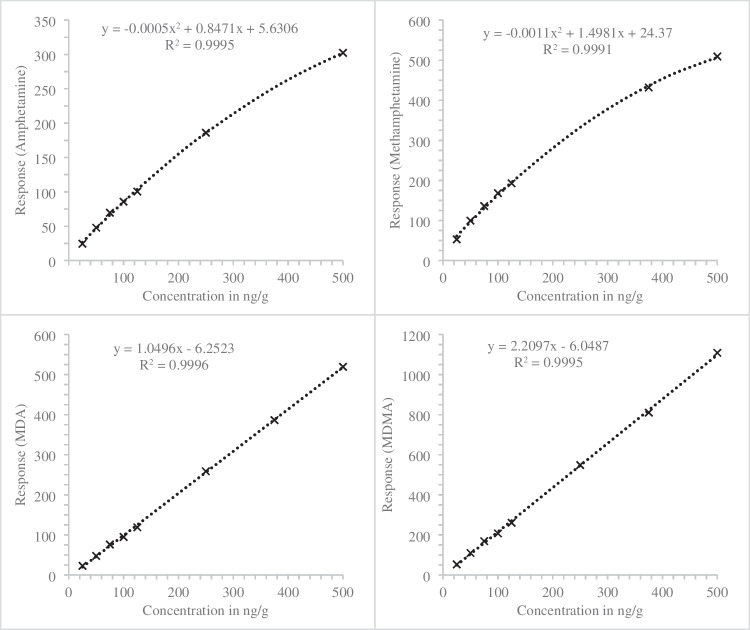
Calibration curve of amphetamine (quadratic), methamphetamine (quadratic), MDA (linear), and MDMA (linear) in pig brain (*n* = 2) after fluid–fluid-extraction with the respective regression coefficients (*R*^2^) and calibration functions

The linearity of analytical measurements was established for methylenedioxyamphetamine (MDA) and methylenedioxymethamphetamine (MDMA) within a concentration range of 25 to 750 ng/ml in serum. Conversely, amphetamine and methamphetamine necessitated determination via quadratic calibration curves, within a narrower range of 25 to 625 ng/ml in serum. Figure 7 illustrates the calibration curves along with the respective curve functions and regression coefficients employed for quantification of these substrates in serum samples. Additionally, for the quantification of amphetamine and methamphetamine in tissue samples, quadratic calibration was employed, whereas MDA and MDMA were quantified linearly (refer to Fig. 8).

A total of 42 serum samples obtained from healthy individuals, along with 26 post-mortem brain and 28 post-mortem liver samples, were analyzed with regard to the TIQs 1,3-diMeTIQ, *N*-Me-1,3-diMeTIQ, 1,3-diMe-7,8-MDTIQ, and *N*-Me-1,3-diMe-7,8-MDTIQ as well as their substrates. Table 8 and Table 9 present the concentrations of amphetamine and its derivatives in serum samples from living subjects as well as from post-mortem brain and liver tissue. In addition, blood alcohol concentrations (BAC) are summarized in the tables, ranging from 0.31‰ (sample 8) to 3.44‰ (sample 15). Notably, five cases exhibited BAC values below 0.5‰ (cases 8, 17, 21, 26, and 34), while 24 samples recorded BAC levels exceeding 1‰. Among the 42 serum samples analyzed, 36 were tested positive for amphetamine, with methamphetamine identified in 31 cases. The highest concentration of amphetamine was found in case 26 (542 ng/ml). Furthermore, MDMA was identified in 17 cases, 14 of which were also positive for MDA.

**Table 8:** Concentrations of amphetamine, methamphetamine, MDA, and MDMA in serum in ng/ml. The results of blood alcohol concentration (BAC) in ‰ (g/kg) were obtained from routine analysis

Case	c(amphetamine) in ng/ml	c(methamphetamine) in ng/ml	c(MDA) in ng/ml	c(MDMA) in ng/ml	BAC in ‰
1	111	Negative	Negative	Pos., n. q	1.04
2	Negative	Negative	~ 6^a^	47	0.97
3	Negative	Negative	~ 2^a^	~ 12^a^	1.78
4	40	Pos., n. q	Negative	Negative	1.03
5	96	Pos., n. q	Negative	Negative	1.49
6	353	Pos., n. q	~ 10^a^	36	0.51
7	~ 20^a^	Pos., n. q	Negative	Negative	1.31
8	487	Pos., n. q	Pos., n. q	Pos., n. q	0.31
9	~ 6^a^	Negative	Negative	Negative	0.80
10	227	Pos., n. q	Negative	Negative	1.37
11	31	Pos., n. q	Negative	Negative	1.27
12	~ 10^a^	Pos., n. q	Negative	Negative	1.93
13	61	Pos., n. q	Negative	Negative	1.39
14	45	Negative	Negative	Negative	1.05
15	44	Pos., n. q	Negative	Negative	3.44
16	39	Pos., n. q	Negative	Negative	1.95
17	341	Pos., n. q	~ 10^a^	100	0.33
18	Negative	Negative	~ 6^a^	94	1.58
19	Pos., n. q	Negative	~ 10^a^	205	0.56
20	Negative	Negative	~ 10^a^	84	1.74
21	77	Pos., n. q	Negative	Negative	0.4
22	219	Pos., n. q	Negative	Negative	0.79
23	Negative	Negative	~ 5^a^	~ 20^a^	0.96
24	58	Pos., n. q	Negative	Negative	0.57
25	54	Pos., n. q	Negative	Pos., n. q	1.08
26	542	Pos., n. q	Negative	Negative	0.49
27	55	Pos., n. q	Negative	Negative	0.55
28	401	Pos., n. q	Negative	Negative	1.34
29	43	Pos., n. q	Negative	Negative	1.87
30	89	Pos., n. q	~ 3^a^	~ 10^a^	0.74
31	Negative	Negative	~ 10^a^	153	1.06
32	139	Pos., n. q	Pos., n. q	Pos., n. q	0.57
33	229	Pos., n. q	Negative	Negative	0.77
34	157	Pos., n. q	~ 3^a^	~ 10^a^	0.38
35	352	Pos., n. q	Negative	Negative	0.83
36	114	Pos., n. q	Negative	Negative	1.22
37	45	Pos., n. q	Pos., n. q	Pos., n. q	1.17
38	81	Pos., n. q	Negative	Negative	1.15
39	45	Pos., n. q	Negative	Negative	1.12
40	97	Pos., n. q	Negative	Negative	0.89
41	50	Pos., n. q	Negative	Negative	1.54
42	115	Pos., n. q	Negative	Pos., n. q	1.26

^a^The concentration is below the calibration range; ~ , approximately; *Pos., n. q.*, positive, not quantified

**Table 9:** Concentrations of amphetamine, methamphetamine, MDA, and MDMA in human liver and brain in ng/g. The results of blood alcohol concentration (BAC) in ‰ (g/kg) were obtained from routine analysis

Case	Matrix	c(amphetamine) in ng/g	c(methamphetamine) in ng/g	c(MDA) in ng/g	c(MDMA) in ng/g	BAC in ‰
Bonn 1	Liver	Not available				2.32
	Brain	> 500	Pos., n. q	Negative	Negative	
Duesseldorf 1	Liver	> 500	Pos., n. q	~ 120	~ 3200^a^	0.12
	Brain	> 500	Pos., n. q	~ 80	~ 830^a^	
Duesseldorf 2	Liver	~ 150	Negative	Pos., n. q	~ 30	2.23
	Brain	~ 140	Pos., n. q	Negative	Negative	
Duesseldorf 3	Liver	Pos., n. q	Pos., n. q	~ 140	~ 4100^a^	1.68
	Brain	> 500	Negative	~ 40	~ 2300^a^	
Duesseldorf 4	Liver	> 500	~ 30	Negative	~ 20	1.19
	Brain	> 500	~ 30	Negative	Negative	
Duesseldorf 5	Liver	Pos., n. q	Negative	~ 690^a^	~ 5400^a^	1.35
	Brain	Negative	Negative	~ 320	~ 4600^a^	
Duesseldorf 6	Liver	> 500	Pos., n. q	Pos., n. q	~ 30	0.65
	Brain	> 500	Pos., n. q	Negative	Negative	
Essen 1	Liver	> 500	Pos., n. q	~ 240	~ 3100^a^	< 0.1
	Brain	> 500	Pos., n. q	~ 260	~ 6500^a^	
Essen 2	Liver	> 500	Pos., n. q	Pos., n. q	Pos., n. q	0.25
	Brain	~ 400	Pos., n. q	Negative	Negative	
Essen 3	Liver	> 500	Pos., n. q	Negative	Pos., n. q	0.17
	Brain	> 500	Negative	Negative	Pos., n. q	
Essen 4	Liver	> 500	Pos., n. q	Pos., n. q	Negative	1.11
	Brain	> 500	Pos., n. q	Negative	Pos., n. q	
Essen 5	Liver	Pos., n. q	Negative	~ 160	~ 1100^a^	1.16 (spleen)
	Brain	-	-	-	-	
Essen 6	Liver	> 500	Pos., n. q	~ 50	~ 400	< 0.1
	Brain	> 500	Pos., n. q	~ 20	~ 280	
Essen 7	Liver	> 500	Pos., n. q	~ 30	Pos., n. q	1.22
	Brain	~ 240	Pos., n. q	Negative	Pos., n. q	
Essen 8	Liver	> 500	Pos., n. q	Pos., n. q	Pos., n. q	1.61
	Brain	> 500	Pos., n. q	Negative	Pos., n. q	
Cologne 1	Liver	> 500	Pos., n. q	Pos., n. q	Pos., n. q	1.19
	Brain	~ 420	Pos., n. q	Negative	Pos., n. q	
Cologne 2	Liver	> 500	Pos., n. q	Negative	Pos., n. q	1.99
	Brain	~ 430	Pos., n. q	Negative	Negative	
Cologne 3	Liver	> 500	Pos., n. q	Negative	Pos., n. q	0.62
	Brain	> 500	Pos., n. q	Negative	Negative	
Cologne 4	Liver	~ 40	Negative	~ 480	~ 5700^a^	0.89
	Brain	Pos., n. q	Negative	~ 70	~ 6700^a^	
Cologne 5	Liver	> 500	Pos., n. q	Pos., n. q	~ 30	0.80
	Brain	> 500	Pos., n. q	Negative	Pos., n. q	
Cologne 6	Liver	> 500	Pos., n. q	Negative	Pos., n. q	0.64
	Brain	~ 490	Pos., n. q	Negative	Pos., n. q	
Cologne 7	Liver	Pos., n. q	Negative	~ 300	~ 5300^a^	1.54
	Brain	Negative	Negative	~ 80	~ 3400^a^	
Cologne 8	Liver	> 500	Pos., n. q	Pos., n. q	~ 60	0.41
	Brain	> 500	Pos., n. q	Negative	Pos., n. q	
Cologne 9	Liver	> 500	Pos., n. q	Negative	Pos., n. q	0.73
	Brain	> 500	Pos., n. q	Negative	Negative	
Rostock 1	Liver	> 500	Pos., n. q	Negative	Pos., n. q	1.69
	Brain	> 500	Pos., n. q	Negative	Negative	
Rostock 2	Liver	> 500	Pos., n. q	Negative	Pos., n. q	1.12
	Brain	> 500	Pos., n. q	Negative	Negative	
Rostock 3	Liver	> 500	Pos., n. q	Negative	Pos., n. q	1.72
	Brain	> 500	Pos., n. q	Negative	Negative	
Rostock 4	Liver	> 500	~ 90	Pos., n. q	Pos., n. q	0.51
	Brain	Not available				
Rostock 5	Liver	> 500	Pos., n. q	Pos., n. q	Negative	2.07 (muscle)
	Brain	Not available				

^a^The concentration is out of the calibration range; ~ , approximately; *Pos., n. q.*, positive, not quantified

Quantification of amphetamines in post-mortem brain and liver tissue was achieved through the application of the calibration function outlined in Fig. 8. Amphetamine concentrations surpassing the upper limit of quantification were denoted as > 500 ng/g, owing to the quadratic calibration model. The BAC levels, as determined through routine analysis, ranged from < 0,1‰ up to 2,32‰. Notably, 14 out of 27 samples exhibited BAC values exceeding 1.0‰ (Table 9).

Twenty-four of the 26 brain samples were positive for amphetamine, from which 21 were positive for methamphetamine as well. Among the 15 MDMA-positive cases, concentrations ranged from ~ 280 to 6700 ng/g (case “Essen 6” and “Cologne 4,” respectively), with each MDMA-positive sample also yielding positive MDA results.

Liver and brain samples of the same origin were listed together. Except for six liver samples, all amphetamine concentrations exceeded the calibration range, thus being declared as > 500 ng/g (Table 9). Methamphetamine was quantified in two cases at concentrations of approximately 30 ng/g and 90 ng/g (“Duesseldorf 4” and “Rostock 4,” respectively). In all other liver samples, methamphetamine was either undetectable or fell just below the lower limit of quantification. In addition, 26 of the 28 liver samples tested were positive for MDMA. Among these, five exhibited concentrations below 100 ng/g (case: “Duesseldorf 2, 4, 6”; “Cologne 5 and 8”), while eight samples recorded MDMA concentrations ranging approximately from 400 to 5400 ng/g (case: “Duesseldorf 1, 3, 5”; “Essen 1, 5, 6”; “Cologne 4 and 7”). Notably, MDA concentrations ranged approximately from 50 to 690 ng/g in these cases.

No trace of TIQs was detected in any serum or brain sample, even in the cases with high substrate concentrations of amphetamines and ethanol (e. g., brain: case “Rostock 1” and “Rostock 3”; serum: cases 10 and 28).

**Fig. 9:**
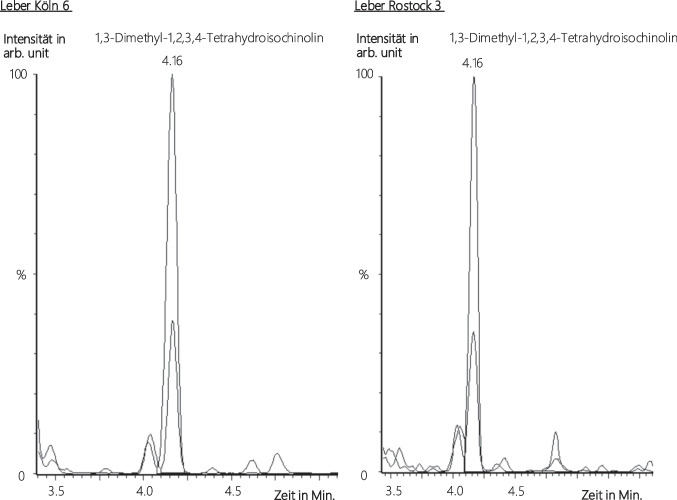
Chromatogram (time in minutes vs. intensity in arbitrary units (arb. unit)) of liver sample “Cologne 6” (left) und “Rostock 3” (right) for *cis*-1,3-diMeTIQ (m/z 162 > 145 and m/z 162 > 117) after fluid–fluid-extraction. Smoothing was accomplished only for the sample “Rostock 3,” because of the small interference in the target mass transition of peak “Cologne 6,” which could be separated in the unsmoothed version

However, liver samples from cases “Cologne 6” and “Rostock 3” yielded positive results for *cis*-1,3-diMeTIQ. Figure 9 presents both MRM transitions (m/z 162 > 145 and 162 > 117) of *cis*-1,3-diMeTIQ to the expected retention time of 4.16 min. Through the calibration function developed during the assessment of the limits of detection (LoD) and lower limits quantification (LLoQ) using Valistad 2.0 software, an estimation of the concentration of 1,3-diMeTIQ was feasible. The calibration function was y = 0.01 x + 0.0041 (*R*^2^ = 0,9997), with (y) as the ratio of the area of the analyte and the ISTD and (x) as the analyte concentration. In case “Cologne 6,” the concentration of 1,3-diMeTIQ was approximately 0.5 ng/g and in case “Rostock 3,” it was approximately 2 ng/g. Both samples exhibited high amphetamine concentrations exceeding 500 ng/g, with corresponding BAC levels of 0.64‰ and 1.72‰, respectively.

## Discussion

The effects of the simultaneous consumption of amphetamine or amphetamine derivatives and alcohol have not yet been adequately clarified, particularly concerning potential neurotoxic condensation products arising from the endogenous reaction of these substances and their metabolites, such as the ethanol metabolite acetaldehyde. 1,3-diMeTIQ, identified as a condensation product of amphetamine and acetaldehyde, has been observed in the brain and blood of chronic alcoholic rats treated with amphetamine or methamphetamine over four weeks. Rats exhibiting abnormal behavior, including tremors, curved posture, stereotyped drooling, hypersensitivity, and genital hypertrophy, showed concentrations of 12.7 ± 2.9 ng/g of 1,3-diMeTIQ in brain. Direct administration of the condensation product to rats resulted in similar or exacerbated reactions, including intense tremors, prostration, or even mortality at higher doses of 1,3-diMeTIQ [4]. Additionally, Makino et al*.* (1990) suggested that 1,3-diMeTIQ decreased the activity of monoamine oxidase (MAO) [4]. Apart from these toxic effects, 1,3-diMeTIQ has exhibited cytotoxicity in SH-SY5Y human neuroblasts and high inhibitory activity towards mitochondrial complex 1 [21]. Several TIQs, including 1,3-diMeTIQ, have been implicated in various toxic effects, such an inhibition of the tyrosine hydroxylase [22, 23], the tryptophan hydroxylase [24], the catechole-*O*-methyltransferase [25], and MAO-A and MAO-B [98], as well as potential involvement in pathogenesis of Parkinson’s disease through biotransformation to an *N*-methylisoquinolinium ion [26–29]. Because of the neurotoxic potential of amphetamine-derived TIQs, the purpose of the present study was to analyze four TIQs, synthesized from amphetamine, methamphetamine, MDMA, and MDA, in human liver, brain tissues, and serum samples. In addition to the brain being the primary site of TIQ action (or effect), liver was also selected because it plays a central role in alcohol metabolism, leading to high levels of the TIQ substrate acetaldehyde compared to other body compartments [30]. Therefore, LC–MS/MS and liquid–liquid-extraction methods were developed and partly validated for the qualitative detection of the aforementioned substances.

The LoDs and LLoQs, determined during method validation, were comparable to literature data. For instance, Musshoff et al. (1999 and 2000) published LoDs for salsolinol and norsalsolinol, condensation products of dopamine and formaldehyde, of 0.2 ng/g and 0.5 ng/g in the human brain using gas chromatography mass spectrometry [31, 32]. Similarly, concentrations of 1,3-diMeTIQ, ranging from 4.5 ± 1.1 to 12.7 ± 2.9 ng/g as reported by Makino et al. (1990), could be detected by the developed method [4] (LoD (*cis*-/*trans*-1,3-diMeTIQ in brain) = 0.3 ng/g; LLoQs (*cis*-/*trans*-1,3-diMeTIQ in brain) = 1.2 and 2.2 ng/g). Furthermore, the method validation showed no evidence of TIQ instabilities even at low concentrations in serum, brain, and liver tissue, suggesting that post-mortem changes during long-term storage or freeze-and-thaw cycles do not affect analyte concentration.

In this study, 42 serum, 26 brain tissue, and 28 liver samples were analyzed. Post-mortem peripheral blood could not be analyzed additionally, as the residual amounts remaining after the routine forensic toxicological analyses served as reserve samples. 1,3-diMeTIQ was found in two liver samples, but only the *cis*-isomers (*cis*-1S,3R- and *cis*-1R,3S-diMeTIQ: t_R_ = 4.16 min) were identified (see Fig. 9). According to Haber et al. (1995), 1,3-diMeTIQ is enzymatically formed rather than under physiological conditions [8]. So far, no specific enzyme responsible for the condensation of amphetamine and acetaldehyde has been identified to date.

Based on the calibration curve used to determine the LLoQ, the concentration of 1,3-diMeTIQ in the two positive liver samples could be estimated—the following results were obtained: “Cologne 6”: c (1,3-diMeTIQ) =  ~ 0.5 ng/g; “Rostock 3”: c (1,3-diMeTIQ) =  ~ 2 ng/g. The amphetamine concentrations in these liver samples exceeded the upper limit of quantification (> 500 ng/g). The BAC levels indicate a strong alcoholization in the “Rostock 3” case (BAC = 1.72‰) and a moderate alcoholization in the “Cologne 6” case (BAC = 0.64‰).

Similar high substrate concentrations were found in 13 other liver samples (“Duesseldorf 4 and 6”; “Essen 4, 7, and 8”; “Cologne 1, 2, 3, 5, and 9”; “Rostock 1, 2, and 5”), but no condensation products could be detected.

The study design of Makino et al. (1990), which includes an 8-weekalcoholism induction in rats [4], suggests that an increased acetaldehyde concentration is crucial for the condensation reaction that can occur in alcoholics. Pilot studies, involving single dose of ethanol or amphetamine, were not described. Furthermore, there is an ongoing debate regarding whether alcoholics exhibits higher acetaldehyde levels than non-alcoholics [33, 34].

Due to limited sample volume, determining the presence of alcohol addiction was not feasible through the analysis of indirect alcohol markers such as γ-glutamyltransferase, mean corpuscular volume, and aspartate and/or alanine aminotransferases [35, 36]. While Harada et al. (1983) reported similar acetaldehyde concentrations (5 µmol/l) in alcoholics and non-alcoholics (3 µmol/l) [33], Palmer and Jenkins (1982) found significantly higher acetaldehyde concentrations in alcoholics (c (acetaldehyde – alcoholics) = 17.8 ± 6 µmol/l; c (acetaldehyde – control) = 10.0 ± 4 µmol/l; *p* < 0.01), despite similar BACs of approximately 1.4‰ in both groups [34]. Consequently, the presence of alcoholism remains uncertain.

Another possible explanation for the occurrence of 1,3-diMeTIQ in only two of 15 cases with similar substrate concentration could be the presence of a deficiency in isozyme I of the aldehyde dehydrogenase (ALDH). Harada et al. (1983) reported mean acetaldehyde concentrations of 30 µmol/l in individuals with ALDH deficiency compared to healthy controls (3 to 5 µmol/l) at an average BAC of 0.5 g/l [33].

Additionally, the absence of TIQs in the aforementioned samples could be attributed to advanced biotransformation of the condensation products, resulting in concentrations below the LoD. Potential metabolic reactions include the following:

1. aromatic hydroxylation [37],
2. oxidative *N*-dealkylation of *N*-Me-1,3-diMeTIQ and *N*-Me-1,3-diMe-7,8-MDTIQ,
3. *O*-demethylation of 7,8-MDTIQs by cytochrome P450 followed by *O*-methylation by catechol-O-methyltransferase,
4. *N*-methylation of 1,3-diMeTIQ, and 1,3-diMe-7,8-MDTIQ [38, 39] followed by oxidation by monoamine oxidase to *N*-methylisochinolinium ion [40], and
5. glucuronidation or sulfatation of the hydroxymetabolites.

This hypothesis also provides a plausible explanation for the non-detection of the *N*-Me-1,3-diMe-7,8-MDTIQ and 1,3-diMe-7,8-MDTIQ in samples exhibiting moderate to high levels of MDMA, MDA, and ethanol (c (MDMA) =  ~ 1100 to 5700 ng/g; c (MDA) =  ~ 140 to 690 ng/g; BAC = 0.89 to 1.60‰). The electron-donating nature of the methylenedioxy substituents in MDMA and their associated positive mesomeric effect lead to an augmentation of electron density within the benzene ring, thereby theoretically facilitating the cyclization reaction [8]. Thus, the absence of acetaldehyde or an advanced metabolism of the TIQs is the sole conceivable explanation for the absence of any MDMA- or MDA-condensation product in this investigation.

In all other liver samples (“Duesseldorf 1, 2”; “Essen 1, 2, 3; 6”; “Cologne 8”; “Rostock 4”), it appears that either the concentration of one of the substrates is insufficient for the condensation reaction, or the TIQ concentration is marginally below the LoD.

In brain tissue and serum, no TIQs were detected, irrespective of substrate concentration. This could be explained by the majority of acetaldehyde production occurring in the liver compared to cerebral or peripheral blood. Moreover, 95% of liver acetaldehyde is metabolized to acetic acid, with only 5% entering the bloodstream. Eriksson and Sippel (1977) published negative results for acetaldehyde in brain tissue [168]. Further studies have reported that only with saturated ALDHs present in cerebral capillaries acetaldehyde can enter the brain for potential condensation with amphetamines (c (acetaldehyde) = 250 nmol/ml or 70 µM [169; 170]). Alternatively, the absence of TIQs in brain tissue and blood samples could be explained by the condensation reaction occurring exclusively in the liver, resulting in dilution of TIQs upon subsequent distribution to the bloodstream and brain. For the detection of 1,3-diMe-TIQ in blood and brain in case “Cologne 6” and “Rostock 3,” a more sensitive method with lower LoDs would be necessary.

## Conclusion

For the detection of the condensation products 1,3-diMeTIQ, *N*-Me-1,3-diMeTIQ, 1,3-diMe-7,8-MDTIQ, and *N*-Me-1,3-diMe-7,8-MDTIQ and their amphetamine substrates in blood, brain, and liver tissue, a more sensitive method with lower LoDs would be necessary.

## Supplementary Information

Below is the link to the electronic supplementary material.

Supplementary file1 (DOCX 6555 KB)

## Abbreviations

1,3-diMeTIQ: 1,3-Dimethyl-1,2,3,4-tetrahydroisoquinoline
1,3-diMe-7,8-MDTIQ: 1,3-Dimethyl-7,8-methylenedioxy-1,2,3,4-tetrahydroisoquinoline
90%-CI: 90% confidence interval
ALDH: Aldehyde dehydrogenase
arb. unit: Arbitrary unit
BAC: Blood alcohol concentration
1-(Me-d_3_)-TIQ-d_8_: 1-(Methyl-d_3_)-1,2,3,4-tetrahydroisoquinoline-1,2,3,4,5,6,7,8-d_8_
GTFCh: German Society of Toxicological and Forensic Chemistry
ISTD: Internal standard
LC–MS/MS: Liquid chromatography tandem mass spectrometry
LoD: Limit of detection
LLoQ: Lower limit of quantification
MAO: Monoamine oxidase
MDA: Methylenedioxyamphetamine
MDMA: Methylenedioxymethamphetamine
MRM: Multiple reaction mode
MS: Mass spectrometry
MV: Mean value
N-Me-1,3-diMeTIQ: *N*-Methyl-1,3-dimethyl-1,2,3,4-tetrahydoisoquinoline
N-Me-1,3-diMe-7,8-MDTIQ: *N*-Methyl-1,3-dimethyl-7,8-methylenedioxy-1,2,3,4-tetrahydroisoquinoline
Pos., n. q.: Positive, not quantified
TIQ: Tetrahydroisoquinoline

## Declarations

### Source of biological material

Post-mortem brain and liver samples as well as serum samples of living subjects were obtained from the Institutes of Legal Medicine Bonn, Duesseldorf, Essen, Cologne, and Rostock. For method validation, brain and liver samples, collected during autopsies at the Institute of Legal Medicine Duesseldorf, were used. Sample data were anonymized prior to analysis.
