# Supplementary material for: Analysis of tetrahydroisoquinolines formed after simultaneous consumption of ethanol and amphetamine or its derivatives by LC–MS/MS in human blood, brain, and liver tissue
Source: Anal Bioanal Chem. 2024 Oct 2;416(28):6497–516. doi: 10.1007/s00216-024-05540-1 (PMC11541333; doi:10.1007/s00216-024-05540-1)
Supplement: Supplementary file 1 — Supplementary file1 (DOCX 6555 KB) [file 216_2024_5540_MOESM1_ESM.docx]

**Analysis of tetrahydroisoquinolines formed after simultaneous consumption of ethanol and amphetamine or its derivatives by LC-MS/MS in human blood, brain and liver tissue**

**Marianne Sonnenberg^1*^, Constantin Czekelius^2^, Oliver Temme^1^, Evelyn Pawlik^1^, Thomas Daldrup^1^**

^1^Institute of Legal Medicine, University Hospital Düsseldorf, Moorenstraße 5, 40225 Düsseldorf, Germany

^2^Institut of Organic Chemistry and Macromolecular Chemistry, Heinrich-Heine-University Duesseldorf, Universitätsstraße 1, 40225 Düsseldorf, Germany

*Corresponding author: fortoxi@uni-duesseldorf.de

**
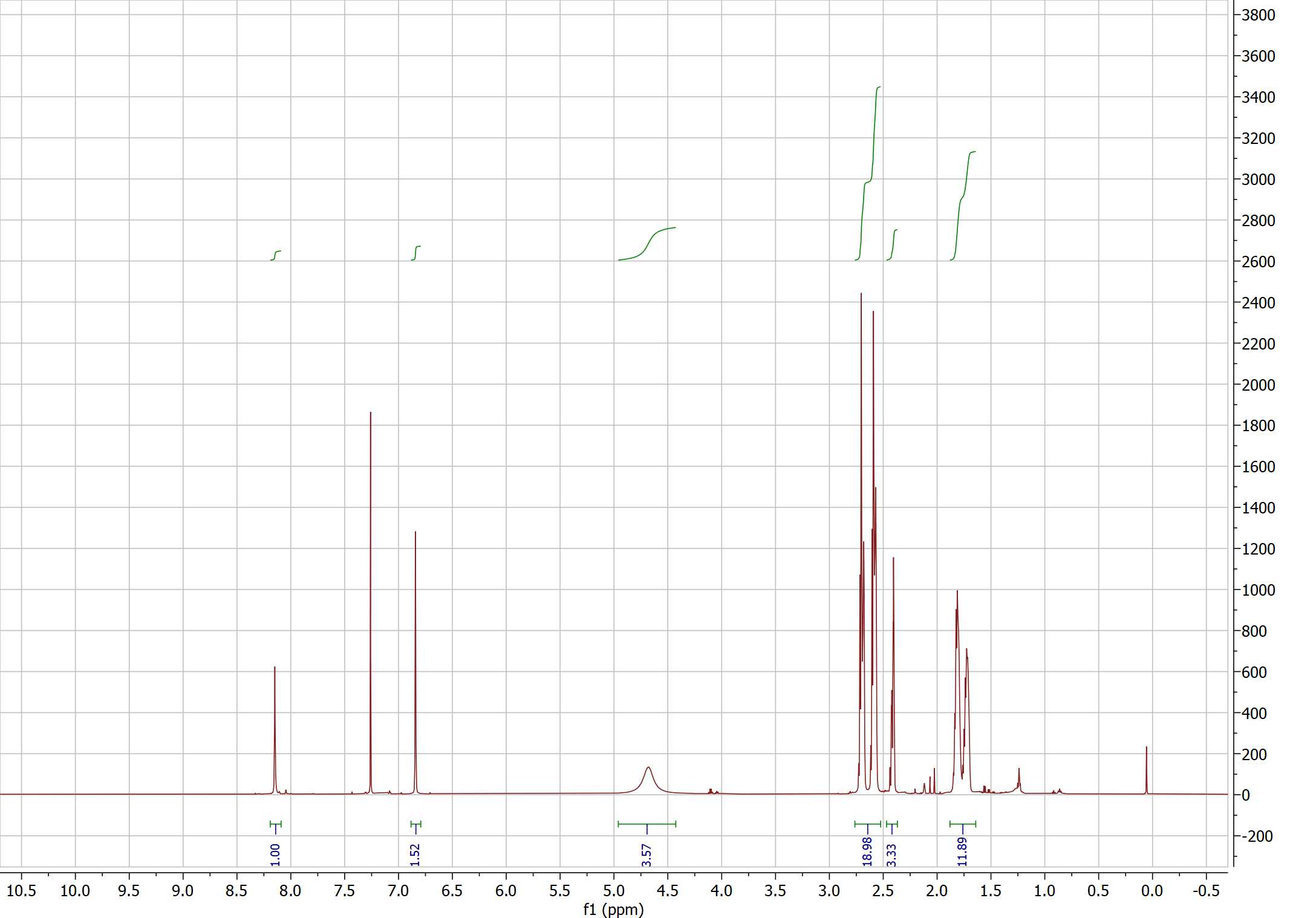
**

**Fig. S1** 1-(Methyl-d_3_)-1,2,3,4-tetrahydroisoquinoline-1,2,3,4,5,6,7,8-d_8_ (^1^H NMR, CDCl_3_, 600 MHz)


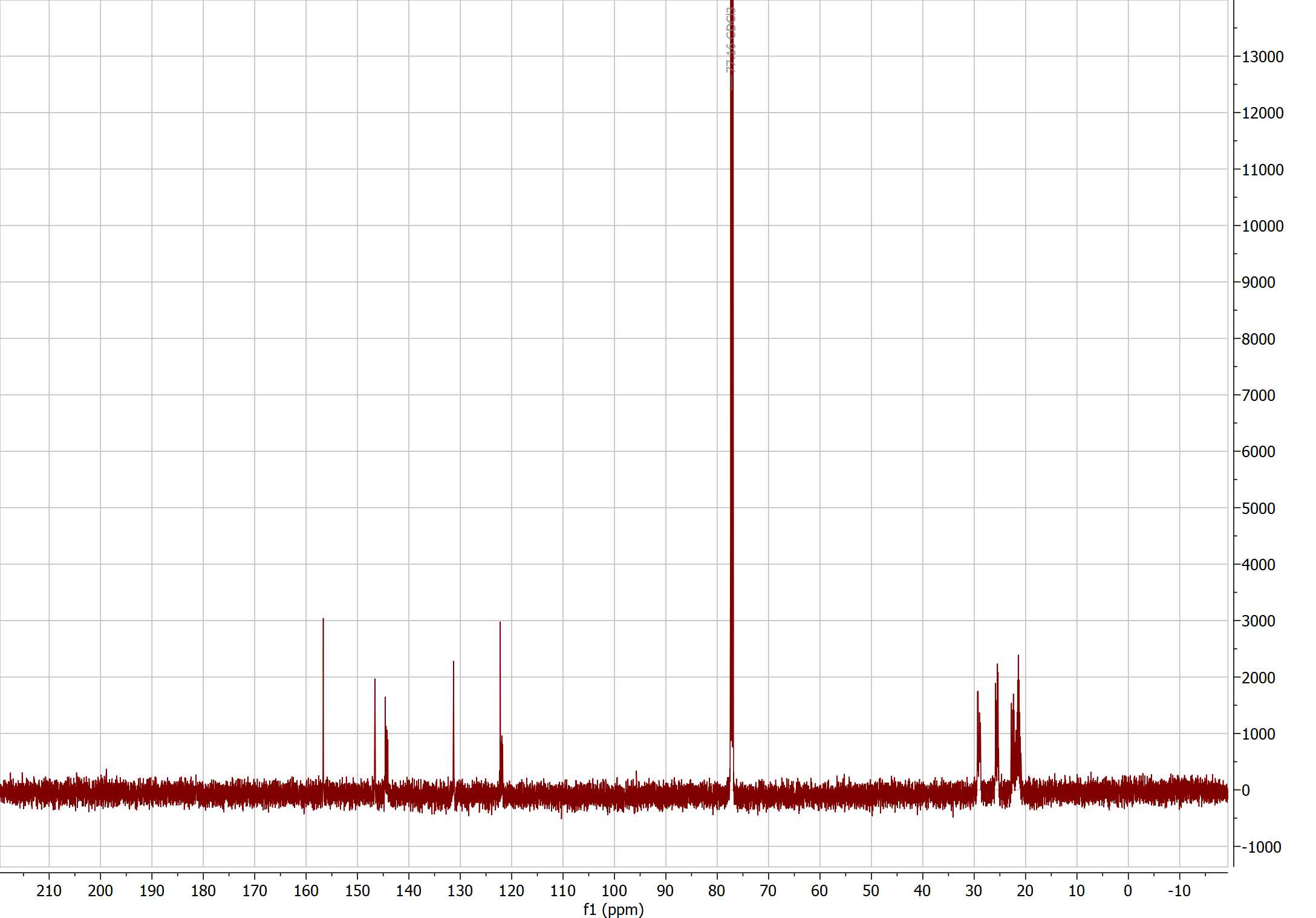


**Fig. S2** 1-(Methyl-d_3_)-1,2,3,4-tetrahydroisoquinoline-1,2,3,4,5,6,7,8-d_8_ (^13^C NMR, CDCl_3_, 151 MHz)


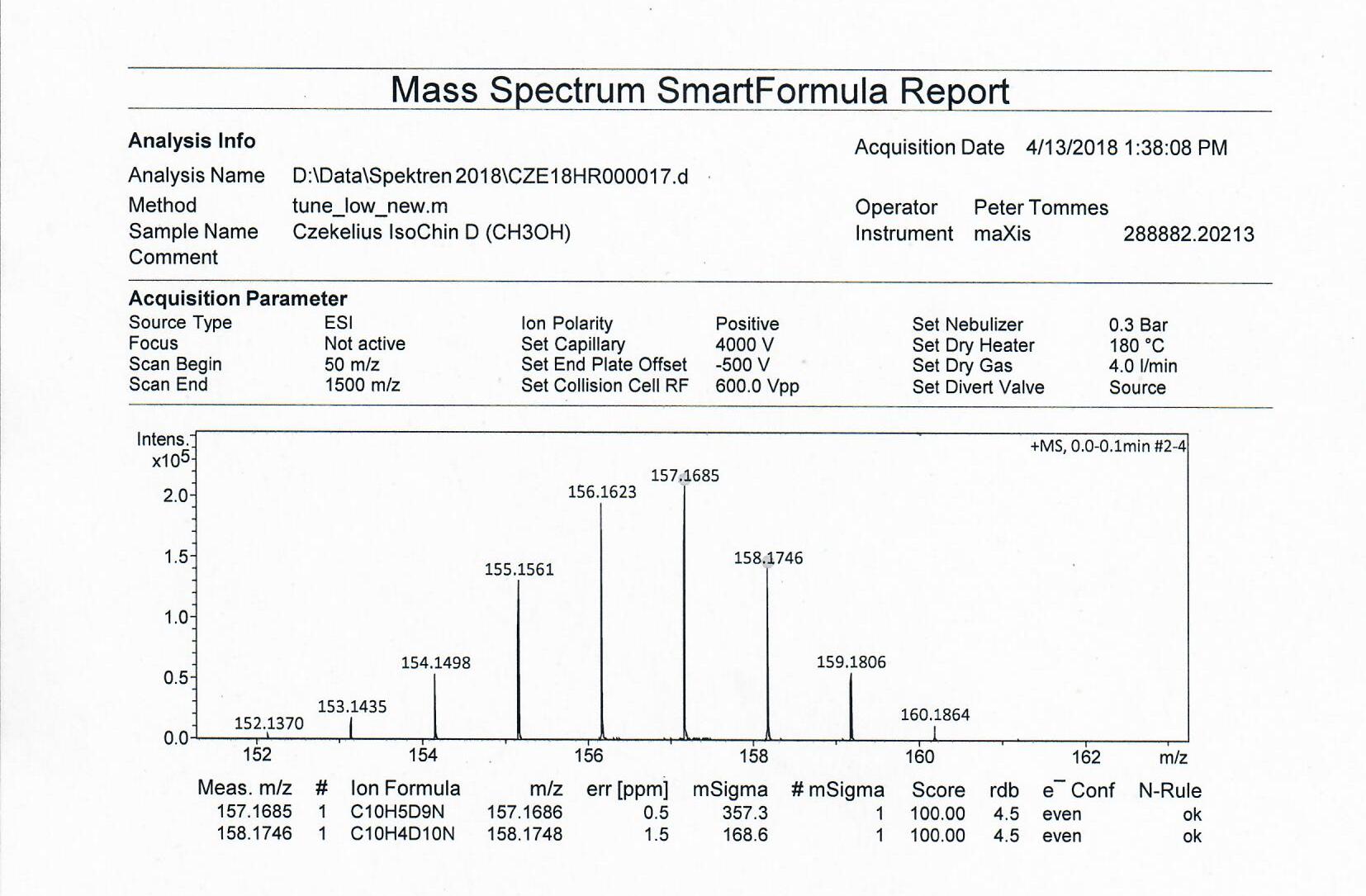


**Fig. S3** 1-(Methyl-d_3_)-1,2,3,4-tetrahydroisoquinoline-1,2,3,4,5,6,7,8-d_8_ (HRMS, ESI, MeOH)

**Fig. S4** *cis*/*trans*-1,3-Dimethyl-1,2,3,4-tetrahydroisoquinoline (^1^H-NMR, CDCl_3_, 600 MHz)

**

**Fig. S5** *trans*-1,3-Dimethyl-1,2,3,4-tetrahydroisoquinoline (^1^H-NMR, CDCl_3_, 600 MHz)

*
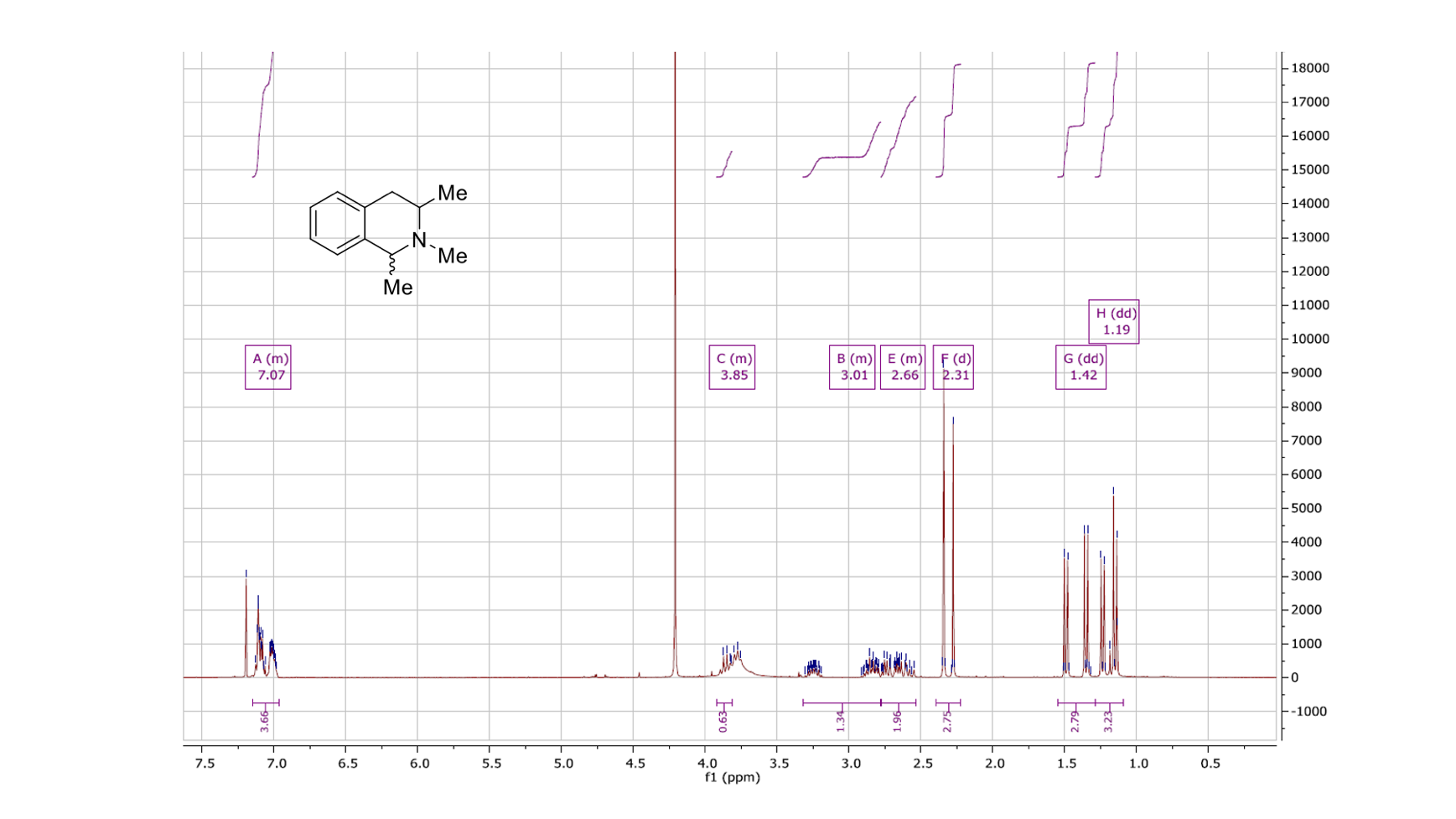
*

**Fig. S6** *cis*/*trans*-1,2,3-Trimethyl-1,2,3,4-tetrahydroisoquinoline (^1^H-NMR, CDCl_3_, 600 MHz)

**
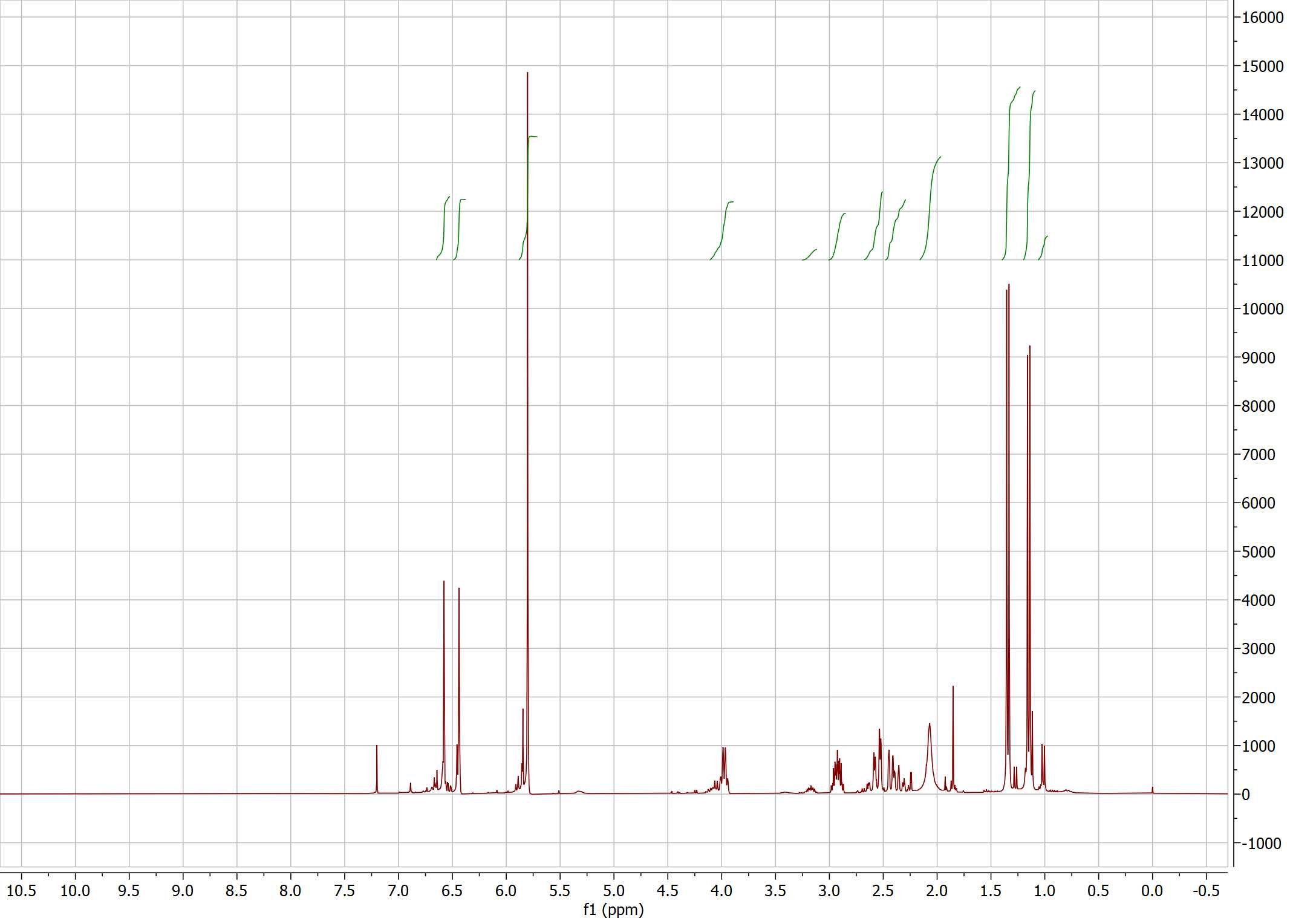


**Fig. S7** *cis*/*trans*-5,7-Dimethyl-5,6,7,8-tetrahydro-[1,3]dioxolo[4,5-*g*]isoquinoline (^1^H-NMR, CDCl_3_, 300 MHz)

**
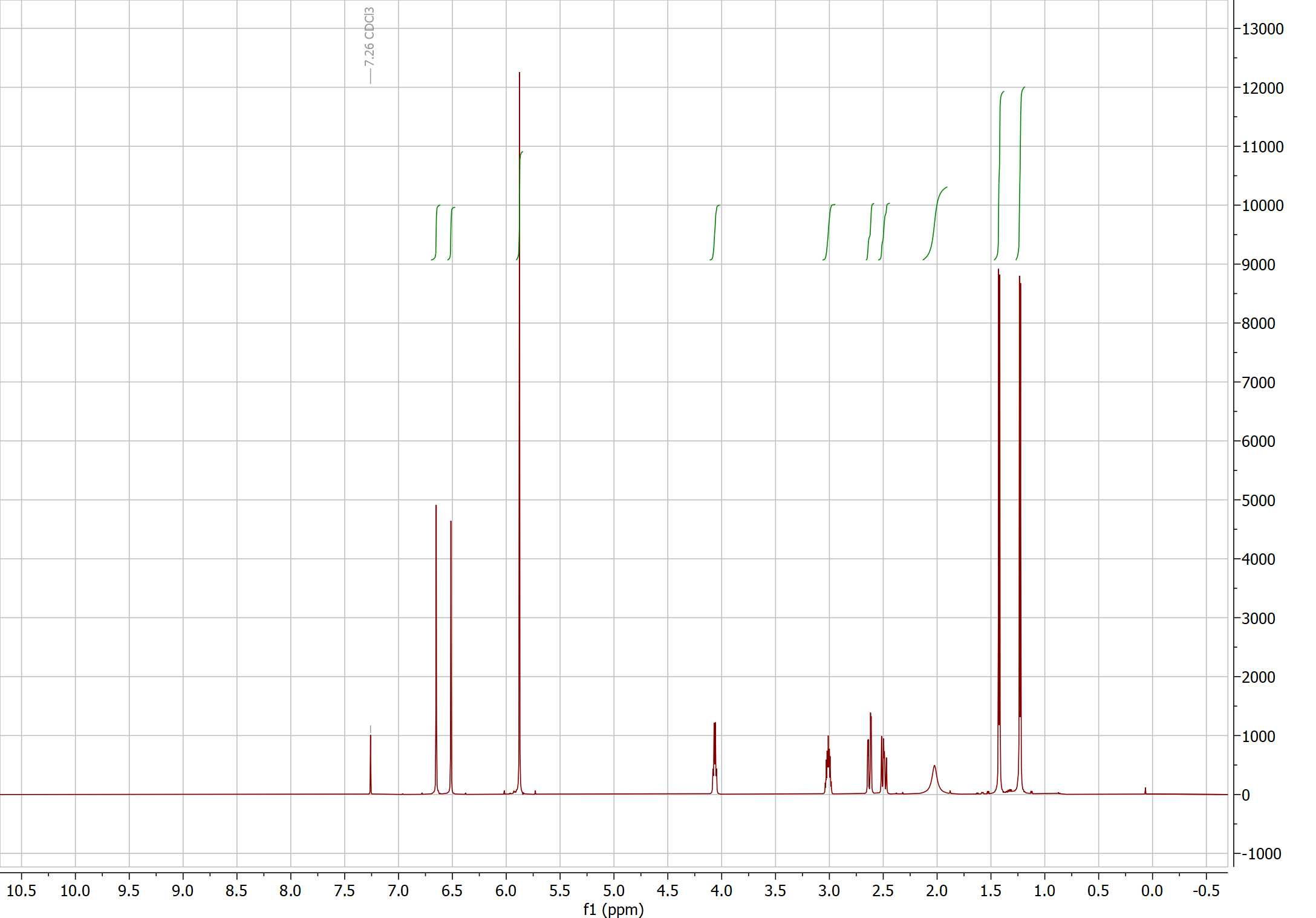


**Fig. S8** *trans*-5,7-Dimethyl-5,6,7,8-tetrahydro-[1,3]dioxolo[4,5-*g*]isoquinoline (^1^H-NMR, CDCl_3_, 600 MHz)


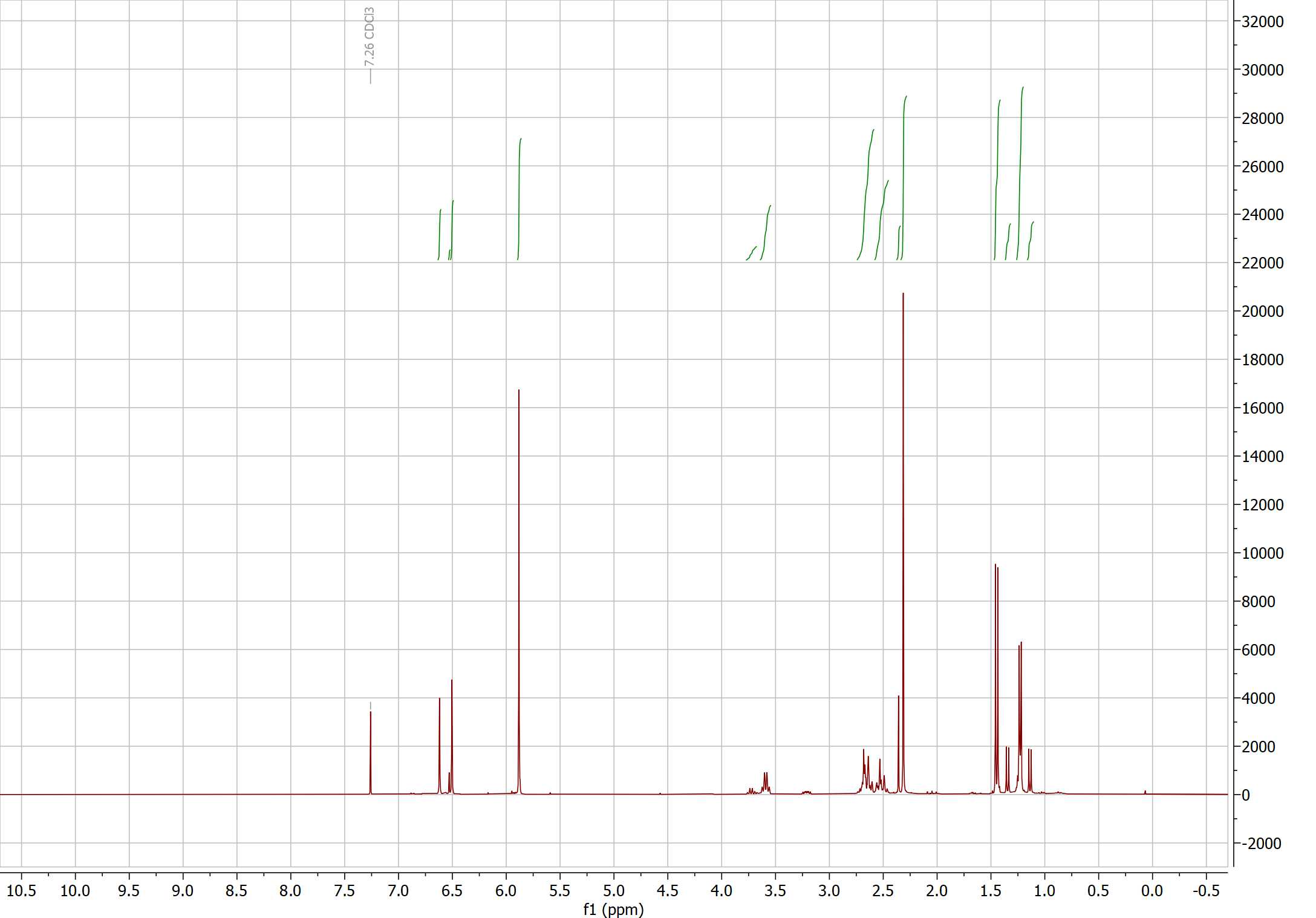


**Fig. S9** *cis*/*trans*-5,6,7-Trimethyl-5,6,7,8-tetrahydro-[1,3]dioxolo[4,5-*g*]isoquinoline (^1^H-NMR, CDCl_3_, 600 MHz)


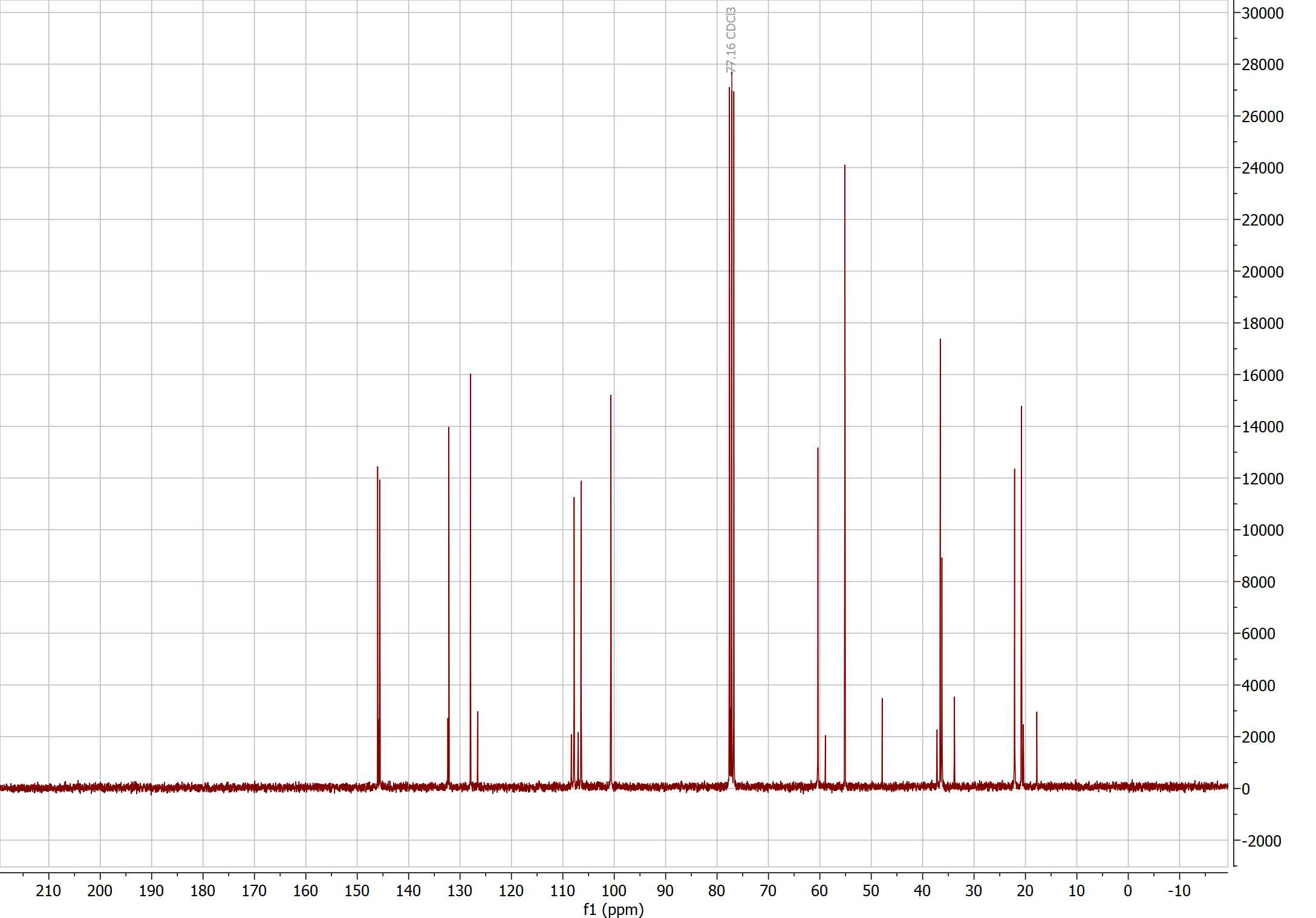


**Fig. S10** *cis*/*trans*-5,6,7-Trimethyl-5,6,7,8-tetrahydro-[1,3]dioxolo[4,5-*g*]isoquinoline (^13^C-NMR, CDCl_3_, 151 MHz)
